# Genome-wide association study for in vitro digestibility and related traits in triticale forage

**DOI:** 10.1186/s12870-024-04927-7

**Published:** 2024-03-27

**Authors:** Anneleen De Zutter, Maria Chiara Piro, Steven Maenhout, Hans Peter Maurer, Johan De Boever, Hilde Muylle, Isabel Roldán-Ruiz, Geert Haesaert

**Affiliations:** 1 Faculty of Bioscience Engineering, Department of Plants and Crops, Ghent University, Diepestraat 1, Bottelare, 9820 Belgium; 2 Faculty of Bioscience Engineering, Department of Plants and Crops, Ghent University, Coupure Links 653, Ghent, 9000 Belgium; 3 State Plant Breeding Institute, University of Hohenheim, 70599 Stuttgart, Germany; 4 Animal Sciences Unit, Flanders Research Institute for Agriculture, Fisheries and Food (ILVO), Scheldeweg 68, Melle, 9090 Belgium; 5 Plant Sciences Unit, Flanders Research Institute for Agriculture, Fisheries and Food (ILVO), Caritasstraat 39, Melle, 9090 Belgium

**Keywords:** Triticale, Forage, in vitro digestibility, GWAS, QTL, SNP

## Abstract

**Background:**

Triticale is making its way on dairy farms as an alternative forage crop. This requires the availability of high-yielding triticale varieties with good digestibility. Triticale forage breeding mainly focussed on biomass yield, but efforts to improve digestibility are increasing. We previously investigated the interrelationships among different quality traits in soft dough triticale: starch, acid detergent fibre and in vitro digestibility of organic matter (IVOMD) and of neutral detergent fibre (IVNDFD) of the total plant, IVNDFD and Klason lignin of the stems, and ear proportion and stem length. Here we determine the genetic control of these traits, using a genome-wide association (GWAS) approach. A total of 33,231 DArTseq SNP markers assessed in a collection of 118 winter triticale genotypes, including 101 varieties and 17 breeding lines, were used.

**Results:**

The GWAS identified a total of 53 significant marker-trait associations (MTAs). The highest number of significantly associated SNP markers (*n* = 10) was identified for total plant IVNDFD. A SNP marker on chromosome 1A (4211801_19_C/T; 474,437,796 bp) was found to be significantly associated with ear proportion, and plant and stem IVNDFD, with the largest phenotypic variation for ear proportion (R²_p_ = 0.23). Based on MTAs, candidate genes were identified which were of particular relevance for variation in in vitro digestibility (IVD) because they are putatively involved in plasma membrane transport, cytoskeleton organisation, carbohydrate metabolic processes, protein phosphorylation, and sterol and cell wall biogenesis. Interestingly, a xyloglucan-related candidate gene on chromosome 2R, SECCE2Rv1G0126340, was located in close proximity of a SNP significantly associated with stem IVNDFD. Furthermore, quantitative trait loci previously reported in wheat co-localized with significantly associated SNP markers in triticale.

**Conclusions:**

A collection of 118 winter triticale genotypes combined with DArTseq SNP markers served as a source for identifying 53 MTAs and several candidate genes for forage IVD and related traits through a GWAS approach. Taken together, the results of this study demonstrate that the genetic diversity available in this collection can be further exploited for research and breeding purposes to improve the IVD of triticale forage.

**Supplementary Information:**

The online version contains supplementary material available at 10.1186/s12870-024-04927-7.

## Background

Triticale (x *Triticosecale* Wittmack) is the intergeneric hybrid of wheat (*Triticum* spp.) and rye (*Secale* spp.) [1, 2] and is mostly used as feed grain [3]. In addition to its great grain yield potential, triticale also possesses interesting high-yielding properties as a forage crop [4, 5]. With the expectation of more frequent dry summers in Western Europe, winter triticale becomes an attractive alternative to maize (*Zea mays* L.), a well-established forage crop on dairy farms [6–9]. Consequently, the primary focus of triticale forage breeding was the development of high-yielding varieties [10]. Nevertheless, triticale forage quality (FQ) is generally inferior to that of maize forage and research to improve its FQ is lagging behind that of other forage crops [4, 11]. Given the rising interest in triticale forage nowadays, a shift towards the improvement of its FQ is taking place in several breeding programmes, such as that at the Field Crop Development Centre (FCDC) in Canada [5, 12, 13].

FQ is a complex trait, that is highly influenced by the biochemical composition and digestibility of the plant [14]. The organic matter digestibility of forage is usually estimated in vitro (abbreviated as IVOMD) and is largely determined by its starch (STA) and fibre content [15]. It is well-known that the fibre fraction is a key determinant of FQ [16]. The fibre or cell wall (CW) fraction is commonly referred to as neutral detergent fibre (NDF), which is composed of cellulose (CELL), hemicellulose (HCELL), and lignin. The content of CELL and lignin are together measured as acid detergent fibre (ADF) [17]. Micro-organisms in the rumen can partially digest NDF and ADF whereas the presence of lignin inhibits the cell wall digestibility (CWD) [16–18]. Moreover, it is important to take into account that the digestibility of the plant and its different parts largely depends on the genotype and the stage in plant development [5, 19–22]. For example, for small grain forages harvested at the soft dough stage, in vitro digestibility *(*IVD) of the stems is lower than that of the leaves and the ears [23, 24]. Furthermore, studies in other forage crops such as maize and perennial ryegrass (*Lolium perenne* L.) revealed the strong negative influence of stem Klason lignin (KL) on in vitro neutral detergent fibre digestibility (IVNDFD) [25, 26]. Whereas traditional breeding mainly focuses on the improvement of IVOMD in general, improvement of the CWD or IVNDFD by lowering stem KL could be an interesting breeding goal in triticale forage.

The genetic control of IVD has been extensively investigated in the model plant *Arabidopsis thaliana*, for which quantitative trait loci (QTLs) for IVNDFD were found to be associated with NDF and KL QTLs and with genes annotated as peroxidases [27]. Furthermore, KL QTLs co-localized with candidate genes involved in the lignin monolignol biosynthesis. Lastly, genes related to the biosynthesis of polysaccharide and protein components of the plant CW were also detected in the support interval of NDF and KL QTLs in the study of Barrière et al. [27].

While our current knowledge of the genetic control of IVD and its related traits in triticale is limited, information is available for forage crops such as maize and sorghum (*Sorghum bicolor* (L.) Moench) [18]. In maize (total plant), Méchin et al. [28] reported QTLs for STA, NDF, acid detergent lignin (ADL), and IVNDFD. Several QTLs have also been reported for leaf and stem NDF, ADF, and ADL, comprising genes involved in STA, CELL, and lignin biosynthesis in maize [29–34]. Li et al. [35] and Lorenzana et al. [36] identified QTLs with mostly small effects for glucose, xylose, and KL and for IVNDFD, lignin, CELL, ADF, and NDF, in maize stems. In sorghum, QTLs associated with NDF, ADF, HCELL, CELL, and ADL were detected for the leaf, stem, and total plant fractions [37, 38].

Although genome-wide association studies (GWASs) have been applied in maize, sorghum, and wheat to detect QTLs related to FQ [32–34, 39, 40], to the best of our knowledge, this approach has not been used yet to decipher the genetic control of FQ in triticale. However, several studies related to protein content, grain yield, and plant height highlighted the power of GWAS to detect significant marker-trait associations (MTAs) in triticale [41–43].

This study builds further on the work presented in De Zutter et al. [44], in which the variation in IVD and related subtraits was investigated, using a collection of winter triticale, harvested at the soft dough stage. Phenotypic data obtained from two consecutive growing seasons revealed moderate to high broad-sense heritability of the studied traits (H²: 0.50–0.95) and wide phenotypic variation for IVOMD, IVNDFD, and IVD related subtraits in triticale (coefficient of variation (CV): 3.9–16%). The goal of the presented research is to investigate the genetic control of IVD and related traits in soft dough triticale forage by means of a GWAS. Phenotypic data from a panel of 118 winter triticale genotypes were combined with a set of DArTseq SNP markers in the GWAS.

This article is based on Chap. 5 of the PhD thesis of Anneleen De Zutter [45].

## Methods

### Plant material

The studied triticale collection consisted of 118 winter triticale genotypes and included 101 commercial varieties and 17 breeding lines from European and North American breeding origin. A description of this collection is provided in Supplementary Table 1 and in De Zutter et al. [44]. The European varieties represent seven important triticale breeding origins (Switzerland, Germany, Denmark, France, the Netherlands, Poland, and Romania). The North American varieties originate from Canada and the United States and were specifically bred for forage purposes. The breeding lines were developed by FCDC Lacombe (Canada) and Research Farm Bottelare (Belgium).

### Phenotypic data

The phenotypic data were collected in two field trials that were carried out at the Ghent University’s Research Farm in Bottelare, Belgium (latitude 50°57’43”, longitude 3°45’37”), during the growing seasons 2017–2018 and 2018–2019. The genotypes were sown in microplots (size: 0.75 m²) at a density of 350 kernels/m² and arranged following a randomized complete block design with three replications. All genotypes were harvested as forage at the soft dough maturity stage (GS85, Zadoks scale) [46]. In this study, only the subtraits previously found to be highly correlated with plant IVOMD, plant IVNDFD, and stem IVNDFD (Spearman correlation coefficient ρ > 0.50) [44] are considered, as listed in Supplementary Table 2. These included stem length, ear proportion, total plant STA, total plant ADFom, and stem KL. All compositional and digestibility traits were estimated using near infrared spectroscopy (NIRS) calibration curves specifically developed for soft dough triticale forage [47]. The prediction ability of NIRS was found to be good (ratio of prediction to deviation, RPD ≥ 3.0) for total plant STA, suitable for screening purposes (2.0 ≤ RPD < 3.0) for total plant ADFom, plant IVOMD, plant IVNDFD, and stem IVNDFD, and poor (1.5 ≤ RPD < 2.0) for stem KL.

All statistical analyses were performed in R software (version 4.2, [48]). Phenotypic data were analysed through linear mixed modelling to obtain best linear unbiased predictors (BLUPs) for the genotypes from the combined observations of the two growing seasons [49]. The BLUP value was calculated for each genotype, as described in De Zutter et al. [47; Formula 2]. The resulting genotypic effects were subsequently used as phenotypic input data for the GWAS.

### Quality control of the SNP markers

Single nucleotide polymorphism (SNP) marker data were obtained by a genotyping-by-sequencing (GBS) approach (DArTseq) from Diversity Arrays Technology, Canberra, Australia (https://www.diversityarrays.com/). The initial dataset consisted of 79,121 SNP markers that were aligned to the Wheat_ChineseSpring10 reference genome v1.0 (2018). In a pre-processing step, loci showing more than 20% missing values (CallRate > 0.8) and a minor allele frequency (MAF) of 5% or lower were discarded, using the *gl.filter.callrate* and *gl.filter.maf* functions of the dartR package (version 2.7.2, [50]). In a next step, the k-nearest neighbour algorithm [51] with k = 10 was used to impute the remaining missing SNP calls with the *knncatimpute* function of the scrime package using distance measure “smc” (version 1.3.5, [52]).

### Population structure

Population structure of the triticale collection was inferred using hierarchical clustering. First, the optimal number of genetic clusters was determined using the *find.clusters.genlight* function in the R package adegenet (method: Ward’s hierarchical clustering) (version 2.1.10, [53]). All principal components were retained and options considering one to ten clusters were tested. The optimal number of clusters was determined using the Bayesian information criterion (BIC). Next, hierarchical clustering was performed using the *hclust* function of package stats (version 4.2.1, [54]). To illustrate the population structure in this dataset, a principal component analysis (PCA) plot was generated using the *prcomp* function (stats package) and *ggplot* function (ggplot2 package). In this representation, ellipses were used to depict the identified genetic clusters (groups).

### Association mapping

The GWAS was conducted by the R package GAPIT (version 3) using a Bayesian-information and linkage-disequilibrium iteratively nested keyway (BLINK) model [55] with the default settings. The P*-*values derived from the GWAS were subsequently used to generate Manhattan plots and Quantile-Quantile (Q-Q) plots. Linear regression analysis was used to determine the phenotypic variance explained by each significantly associated SNP marker (R^2^_p_). Therefore, a linear regression between the phenotypic data, the corresponding SNP markers, and the genetic cluster (group) was applied using the *lm* function (stats package). Next, the median BLUP value per genotypic class (0: homozygous reference allele (homozygous REF), 1: homozygous SNP allele (homozygous ALTERNATIVE), 2: heterozygous) of each significant SNP marker was presented.

### Identification of candidate genes

Candidate regions were delineated around the significantly associated SNP markers based on linkage disequilibrium (LD) decay distances (bp). The LD decay distances were estimated based on loci that have been mapped on the A and B subgenomes of the Wheat_ChineseSpring10 reference genome using the *gl.report.ld.map* function in the package dartR. The LD was calculated as the square of the correlation coefficient (r²) between pairs of polymorphic loci [56], and it was assumed that an r² higher than the critical value 0.2 is likely to be caused by genetic linkage [57, 58]. Consequently, the value at which r² is below 0.2 was taken as LD decay distance over a single chromosome. Since triticale does not have a D genome, significantly associated markers mapped to the D genome of wheat together with significantly associated unmapped (UM) markers were BLASTED against the most recent reference rye genome (Secale cereale Lo7 v1 pseudomolecules (2021)) in an attempt to obtain their physical positions on the R subgenome (https://wheat.pw.usda.gov/blast/). A BLASTN with E-value cutoff lower than 1 × 10^− 10^ and an identity of 100% was applied. Due to a low marker density on the R subgenome, the averaged LD decay distance over the A and B subgenomes was considered as the LD decay distance for the R subgenome. Candidate regions refer then to the genomic regions surrounding the significantly associated SNP marker and spanning up to the LD decay distance calculated for the chromosome (subgenome in the case of the R subgenome) where the corresponding SNP marker is located.

A list of genes located in these candidate regions was retrieved from the Wheat Chinese Spring IWGSC RefSeq v1.0 genome assembly (2018) using the ‘GrainGenes Genome Browsers’ (https://wheat.pw.usda.gov/GG3/genome_browser) [59]. In addition, previously identified QTLs in the wheat genome are made available through the Triticeae Toolbox (T3) via the GrainGenes browser [60]. For markers with a successful BLAST hit on the R genome, a list of genes located in the candidate regions was retrieved from the Secale cereale Lo7 v1 pseudomolecules (2021) using the ‘GrainGenes Genome Browsers’ (https://wheat.pw.usda.gov/GG3/genome_browser). For both the wheat and rye reference genomes, molecular function description of the candidate genes and the biological processes in which they are putatively involved was retrieved from Ensembl Plants (https://plants.ensembl.org/Triticum_aestivum/Info/Index, https://plants.ensembl.org/Secale_cereale/Info/Index).

## Results

### Variation in in vitro digestibility and related subtraits

**Fig. 1:**
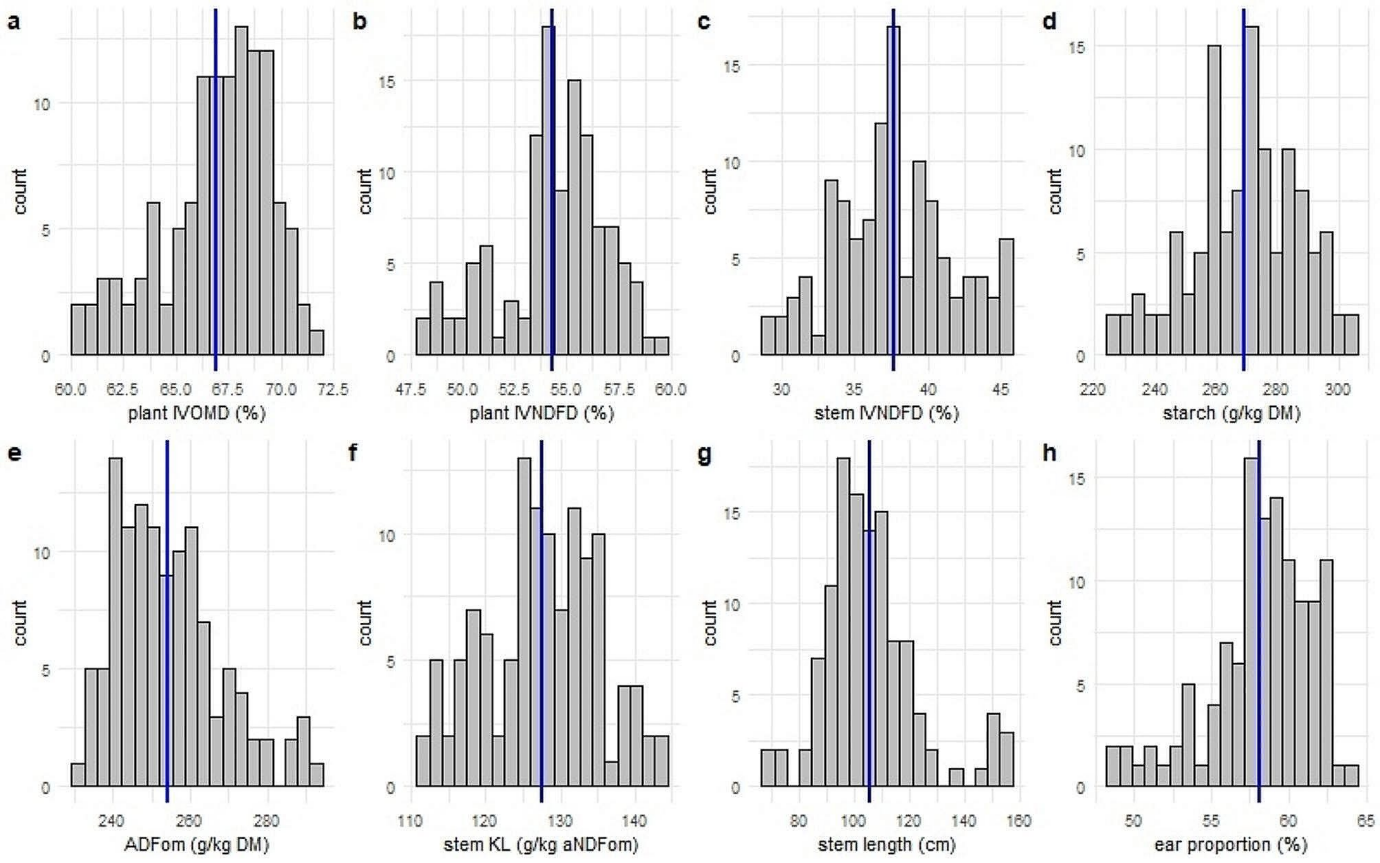
Frequency distribution of the phenotypic traits in this study: total plant in vitro organic matter digestibility (plant IVOMD, **a**), total plant in vitro neutral detergent fibre digestibility (plant IVNDFD, **b**), stem in vitro neutral detergent fibre digestibility (stem IVNDFD, **c**), total plant starch (**d**), total plant acid detergent fibre expressed exclusive of residual ash (ADFom, **e**), stem Klason lignin (stem KL, **f**), stem length (**g**), and ear proportion (**h**). The blue vertical lines indicate the mean

A considerable variation for all investigated traits was observed (Table 1). The CV ranged from 3.9 to 10% for the IVD traits and from 5.4 to 16% for the IVD related subtraits (Table 1). The histograms displayed an approximately normal distribution for all traits (Fig. 1), except for ADFom and stem length for which the distribution was slightly right-skewed, and ear proportion which followed a slightly left-skewed distribution. These data further show suitability for a GWAS approach with H² values between 0.66 and 0.73, and between 0.50 and 0.95 for IVD and related subtraits, respectively (Table 1).

**Table 1:** Summary statistics and broad-sense heritability of the traits considered in the genome-wide association study

Trait (unit)	Mean	SD	MIN	MAX	CV	H²
**IVD traits**						
Plant IVOMD (%)	67	2.6	60	72	3.9	0.66
Plant IVNDFD (%)	54	2.6	48	60	4.7	0.73
Stem IVNDFD (%)	38	3.9	29	46	10	0.72
**IVD related subtraits**						
STA (g/kg DM)	269	18	225	303	6.6	0.50
ADFom (g/kg DM)	254	14	231	293	5.4	0.58
Stem KL (g/kg aNDFom)	128	7.7	111	143	6.0	0.65
Stem length (cm)	106	17	68	154	16	0.95
Ear prop (%)	58	3.3	48	64	5.7	0.76

ADFom, acid detergent fibre expressed exclusive of residual ash; aNDFom, neutral detergent fibre assayed with a heat stable amylase and expressed exclusive of residual ash; CV, coefficient of variation; DM, dry matter; ear prop, ear proportion; H², broad-sense heritability; IVD, in vitro digestibility; IVNDFD, in vitro neutral detergent fibre digestibility; IVOMD, in vitro organic matter digestibility; KL, Klason lignin; MAX, maximum; MIN, minimum; SD, standard deviation; STA, starch

### Quality control of the SNP markers

A total of 33,231 markers were retained after filtering for CallRate and MAF (Additional file 2). The map position was available for 9,029 SNP markers on the A subgenome and for 10,003 SNP markers on the B subgenome (Table 2). About 14,199 of the 33,231 SNP markers (43%) could not be located on the A and B chromosomes in the wheat reference genome. Both mapped and UM SNP markers were used for the GWAS.

**Table 2:** Number of SNP markers and linkage disequilibrium decay distance along chromosomes of the A and B subgenomes

Chr.	Number of SNP markers	LD decay distance (kbp)	Chr.	Number of SNP markers	LD decay distance (kbp)
A subgenome			B subgenome		
1A	1,171	250	1B	1,385	660
2A	1,438	560	2B	1,786	625
3A	1,211	670	3B	1,521	460
4A	990	230	4B	798	400
5A	1,584	480	5B	1,713	420
6A	871	470	6B	1,202	590
7A	1,764	300	7B	1,598	190

The map position was available for 9,029 SNP markers on the A subgenome and for 10,003 SNP markers on the B subgenome. Chr., chromosome; LD, linkage disequilibrium; SNP, single nucleotide polymorphism

### Population structure

**Fig. 2:**
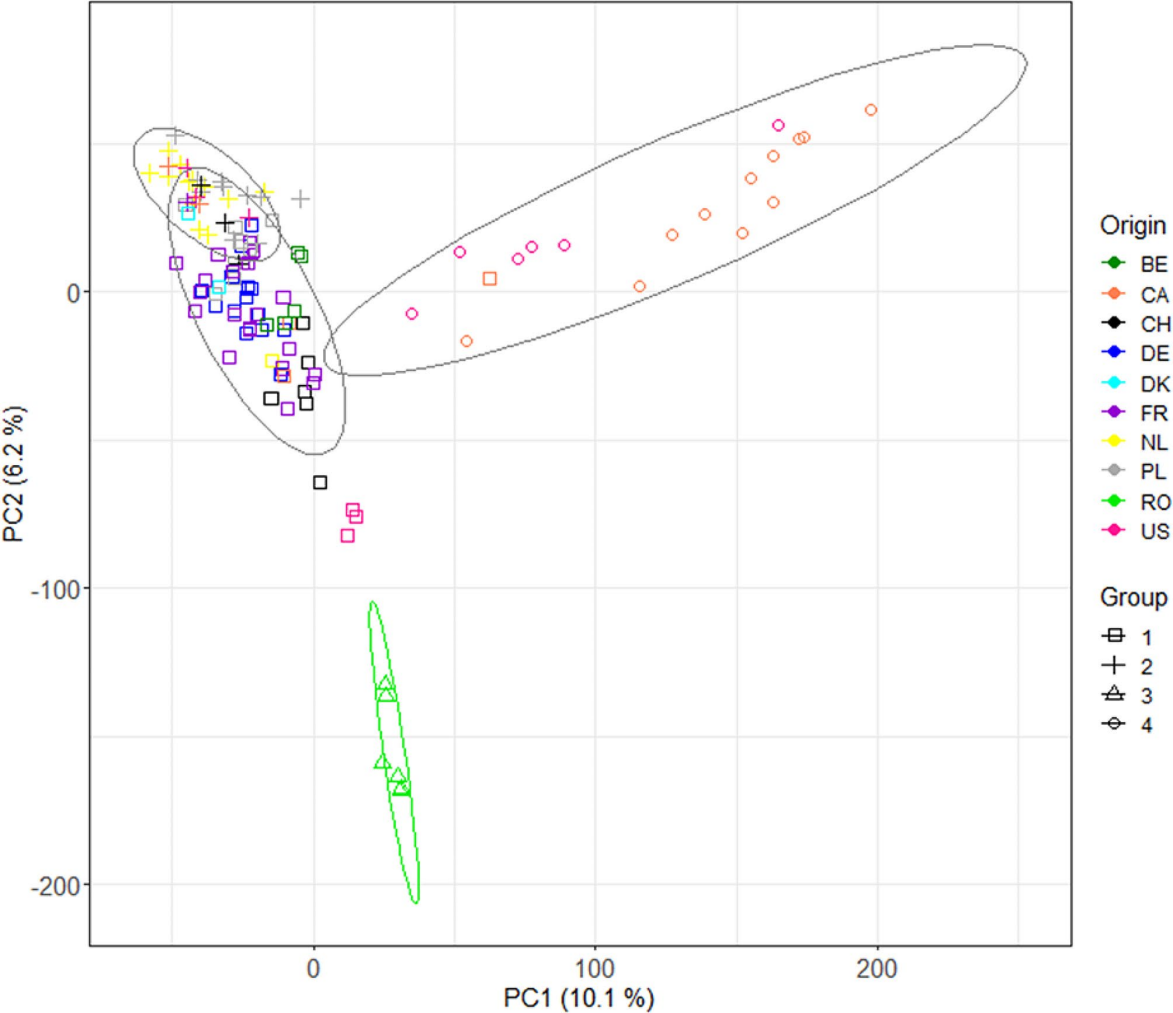
Scatterplot of the first two principal components (PC) showing the four groups. PC1 and PC2 explained 16.3% of the genetic variation in the population. Genotypes are colour-coded based on their country of breeding origin: BE, Belgium; CA, Canada; CH, Switzerland; DE, Germany; DK, Denmark; FR, France; NL, The Netherlands; PL, Poland; RO, Romania, and; US, United States

The optimal number of clusters which minimised the BIC value was four. Subsequently, genotypes were clustered into four groups. However, the first two principal coordinates explained only 16.3% of the total variation (Fig. 2). Hierarchical clustering did not fully follow the classification according to breeding origin (Fig. 2), except for Group 3 which was well-separated from the rest and comprised all six Romanian varieties. Group 1 and 2 are overlapping. Group 1 comprised 63 genotypes and was largely dominated by genotypes from French and German origin. Group 2 was composed of 32 genotypes and differed from Group 1 in the composition of its European subset, which was dominated by Polish and Dutch varieties. Lastly, Group 4 was composed of 17 North American genotypes.

### GWAS and identification of candidate genes

LD decay distances were in the range of 230 kbp to 670 kbp for chromosomes of the A subgenome and in the range of 190 kbp to 660 kbp for chromosomes of the B subgenome (Table 2). The LD decay distance was 625 kbp and 470 kbp over the A and B subgenome, respectively. The averaged LD decay distance over the A and B subgenomes was thus 550 kbp.

**Fig. 3:**
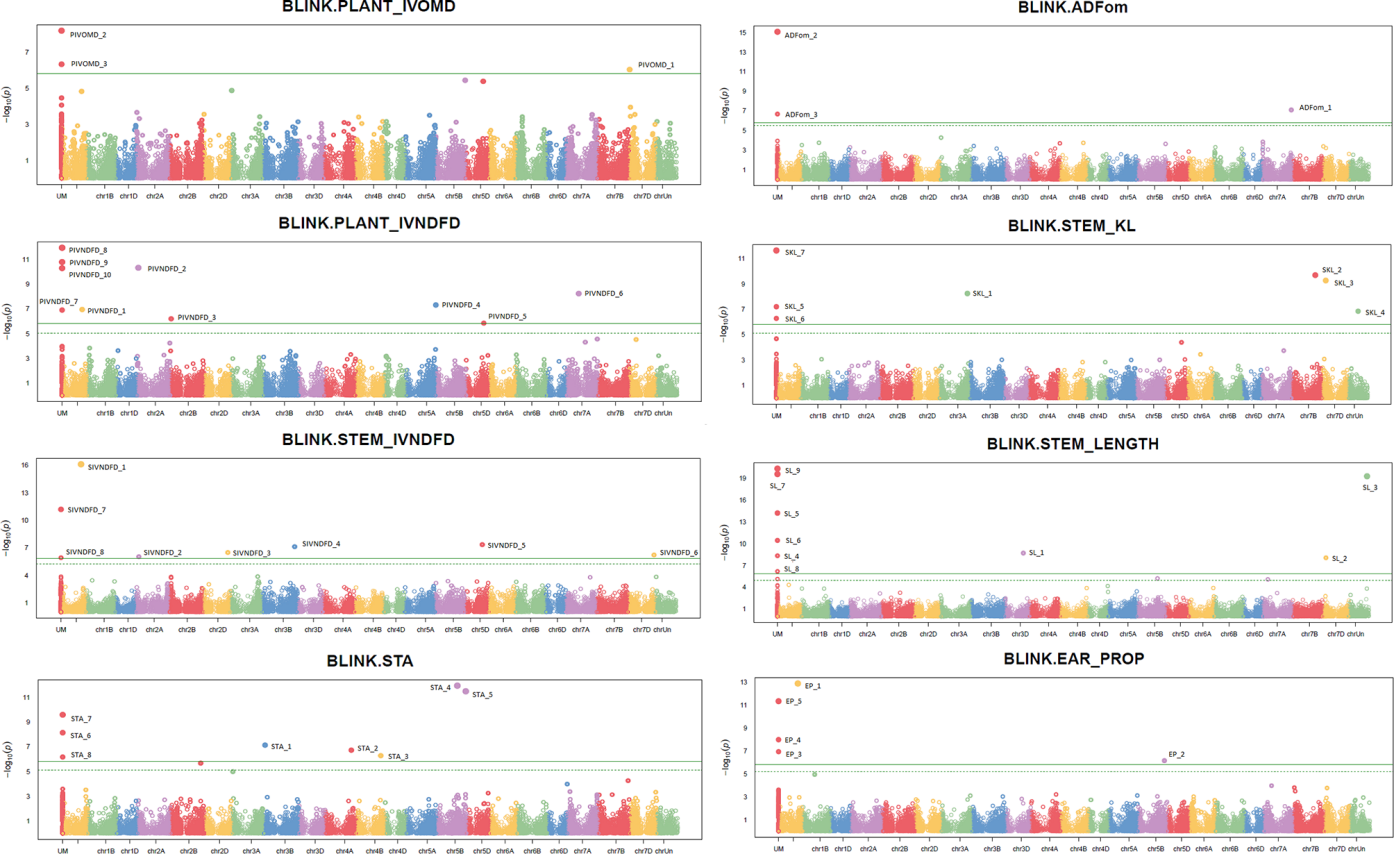
Manhattan plots of the GWAS for the different traits: total plant in vitro organic matter digestibility (plant_IVOMD), total plant in vitro neutral detergent fibre digestibility (plant_IVNDFD), stem in vitro neutral detergent fibre digestibility (stem_IVNDFD), total plant starch (STA), total plant acid detergent fibre expressed exclusive of residual ash (ADFom), stem Klason lignin (stem_KL), stem length, and ear proportion (ear_prop). The presence of significant markers is illustrated by signal peaks above the significance thresholds. Significant markers are represented by their QTL id. The green dashed line indicates the false discovery rate cutoff line and the green solid line indicates the Bonferroni cutoff line. UM: unmapped SNP markers

**Fig. 4:**
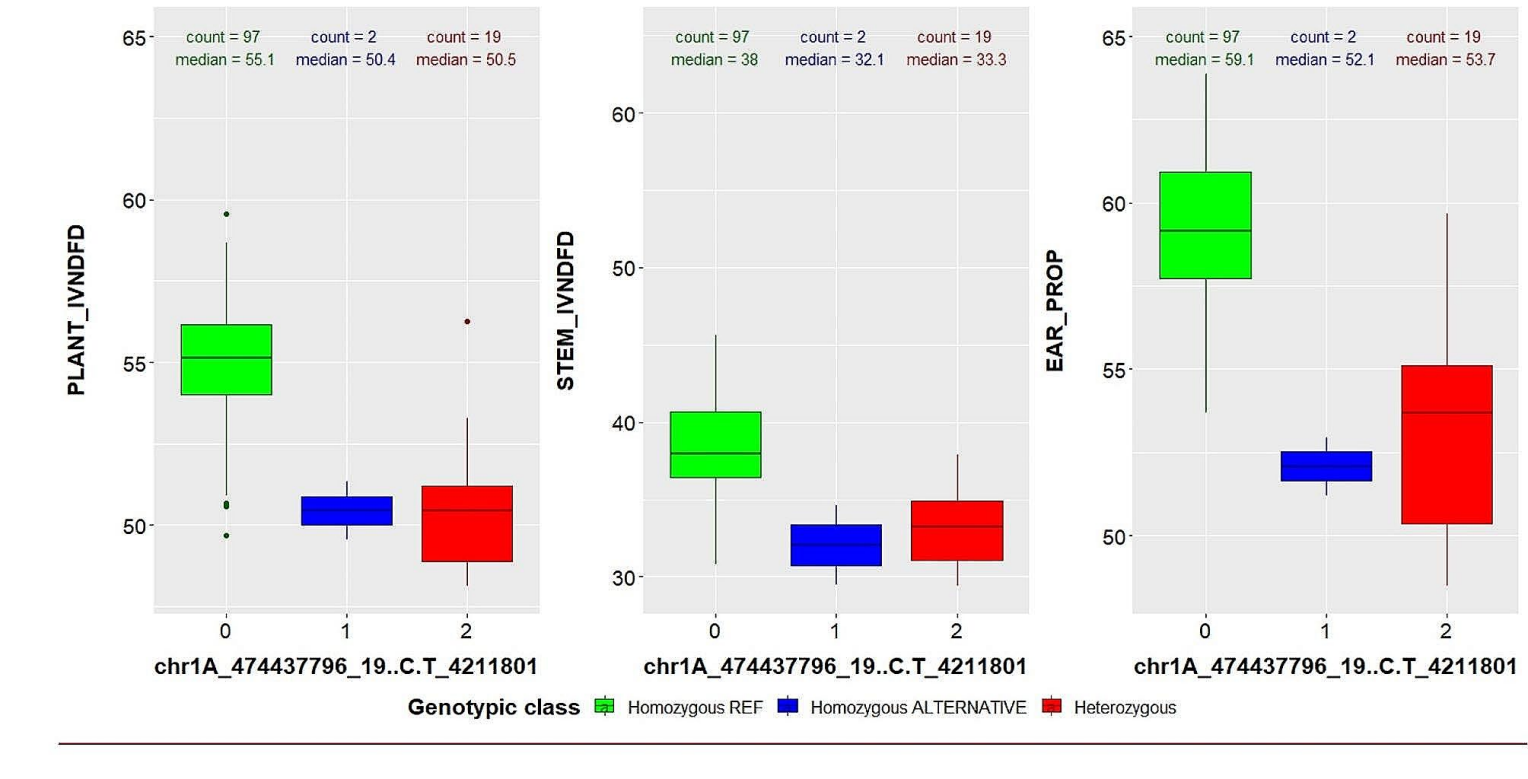
Boxplots showing the variation in phenotype in function of the marker score for SNP marker 4211801_19_C/T on chromosome 1A (474,437,796 bp) significantly associated with more than one trait, where 0: homozygous REF, 1: homozygous ALTERNATIVE and 2: heterozygous. Total plant in vitro neutral detergent fibre digestibility (plant_IVNDFD), stem in vitro neutral detergent fibre digestibility (stem_IVNDFD), and ear proportion (ear_prop)

The results of the GWAS are summarized in Table 3 and in Supplementary Table 3. The GWAS results are represented graphically by the Manhattan plots in Fig. 3. Furthermore, the Q-Q plots are presented in Supplementary Fig. 1. No significant deviation of the distribution of the observed P-values from the distribution of the expected P-values was observed (Supplementary Fig. 1), indicating that the BLINK model sufficiently accounts for population structure and familial relatedness. In total, 53 significant MTAs were identified, of which 10 are located on subgenome A, 8 on subgenome B, 5 on subgenome R, and 30 have UM position (Table 3). Marker 4211801_19_C/T displayed a significant association with more than one trait (Table 3; Fig. 4, and Supplementary Table 3). SNP 4211801_19_C/T is located on chromosome 1A (474,437,796 bp) and is significantly associated with plant IVNDFD, stem IVNDFD, and ear proportion, with the largest phenotypic variation explained for ear proportion (R²_p_ = 0.23) (Table 3). For this marker, triticale plants with a larger ear proportion had as average better IVD characteristics (Fig. 4).

**Table 3:** Results of the genome-wide association study: significantly associated SNP markers with the studied traits (plant IVOMD, plant IVNDFD, stem IVNDFD, STA, ADFom, stem KL, stem length, and ear proportion) and candidate genes in proximity of the significant SNP markers

QTL	SNP marker	Candidate region	P	R²_p_	Median BLUP value per genotypic class (0/1/2)	Candidate gene	Annotation (biological process)
**Plant IVOMD (%)**							
*PIVOMD_1*	*10517787_19_C/T*	*1R:38,333,322–39,433,322*	*6.04*	*0.14*	*67.9/62.6/64.6*	*SECCE1Rv1G0007100*	*Transporter activity, ATP binding, ATP hydrolysis activity*
						*SECCE1Rv1G0007120*	*Peroxidase activity*
PIVOMD_2	22077643_9_T/G	NA	8.20	0.16	NA/62.1/67.7	NA	NA
PIVOMD_3	3042285_21_A/C	NA	6.34	0.11	66.8/65.8/68.3	NA	NA
**Plant IVNDFD (%)**							
PIVNDFD_1	4211801_19_C/T	1A:474,187,796–474,687,796	6.96	0.05	55.1/50.4/50.5	TraesCS1A02G278200	Carbohydrate binding, starch binding
						TraesCS1A02G279000	Hydrolase activity, hydrolyzing O-glycosyl compounds (carbohydrate metabolic process)
PIVNDFD_2	54358850_11_G/A	2A:24,803,946–25,923,946	10.33	0.07	55.6/54.0/53.1	TraesCS2A02G059800	Transmembrane transporter activity (transmembrane transport)
PIVNDFD_3	54354903_15_A/G	2B:18,789,842–20,039,842	6.21	0.13	54.8/51.1/54.6	TraesCS2B02G041200	Methyltransferase activity, O-methyltransferase activity, protein dimerization activity
						TraesCS2B02G041400 TraesCS2B02G041700 TraesCS2B02G042100	UDP-glycosyltransferase activity
						TraesCS2B02G041600	ATPase-coupled transmembrane transporter activity, ABC-type transporter activity (transmembrane transport)
						TraesCS2B02G041800 TraesCS2B02G042000 TraesCS2B02G042200	UDP-glycosyltransferase activity, hexosyltransferase activity
						TraesCS2B02G042300 TraesCS2B02G042400 TraesCS2B02G042900 TraesCS2B02G043000 TraesCS2B02G043100 TraesCS2B02G043200	(exocytosis, protein transport)
PIVNDFD_4	4370557_20_A/G	5A:704,492,970–705,452,970	7.32	0.02	55.4/54.3/54.8	NA	NA
PIVNDFD_5	3623312_68_T/C	NA	5.85	0.01	54.2/52.8/55.9	NA	NA
PIVNDFD_6	4567261_61_T/C	7A:284,227,044–284,827,044	8.24	0.10	NA/54.8/54.1	NA	NA
PIVNDFD_7	14469989_24_A/G	NA	6.91	0.11	54.9/NA/49.2	NA	NA
PIVNDFD_8	15998020_11_A/C	NA	11.95	0.27	NA/57.3/54.1	NA	NA
PIVNDFD_9	22077643_9_T/G	NA	10.80	0.18	NA/49.5/54.9	NA	NA
*PIVNDFD_10*	*3046586_39_G/A*	*5R:609,761,318–610,861,318*	*10.30*	*0.20*	*55.6/50.5/53.7*	*NA*	*NA*
**Stem IVNDFD (%)**							
SIVNDFD_1	4211801_19_C/T	1A:474,187,796–474,687,796	16.12	0.14	38.0/32.1/33.3	See QTL PIVNDFD_1	See QTL PIVNDFD_1
SIVNDFD_2	54345599_25_T/G	2A:62,591,738–63,711,738	6.04	0.10	37.4/37.6/NA	TraesCS2A02G112200	Acyltransferase activity (GPI anchor biosynthetic process)
*SIVNDFD_3*	*4206397_9_C/A*	*2R:844,945,966–846,045,966*	*6.49*	*0.10*	*37.2/39.0/39.6*	*SECCE2Rv1G0126200*	*UDP-glycosyltransferase activity*
						*SECCE2Rv1G0126220*	*ATP binding, ABC-type transporter activity*
						*SECCE2Rv1G0126260 SECCE2Rv1G0126280 SECCE2Rv1G0126290* *SECCE2Rv1G0126300* *SECCE2Rv1G0126310* *SECCE2Rv1G0126320*	*Transmembrane transporter activity* *(transmembrane transport)*
						*SECCE2Rv1G0126340*	*Hydrolase activity, hydrolyzing O-glycosyl compounds*, *xyloglucan:xyloglucosyl transferase activity* *(carbohydrate metabolic process, cellular glucan metabolic process, xyloglucan metabolic process, cell wall biogenesis)*
SIVNDFD_4	10514146_31_A/C	3B:763,613,048–764,533,048	7.13	0.11	37.2/35.8/37.8	NA	NA
SIVNDFD_5	3607057_38_A/G	NA	7.35	0.10	NA/41.9/36.6	NA	NA
SIVNDFD_6	54360481_5_G/T	NA	6.22	0.07	NA/35.9/39.7	NA	NA
SIVNDFD_7	15998020_11_A/C	NA	11.20	0.36	NA/43.2/37.0	NA	NA
SIVNDFD_8	8539528_31_A/G	NA	5.92	0.15	NA/35.1/37.9	NA	NA
**STA (g/kg DM)**							
STA_1	11912016_38_T/C	3B:23,305,225–24,225,225	7.14	0.12	270/259/272	TraesCS3B02G046100	C-5 sterol desaturase activity (sterol biosynthetic process)
						TraesCS3B02G046700	Protein kinase activity (protein phosphorylation)
						TraesCS3B02G046900 TraesCS3B02G047000	Acetylglucosaminyltransferase activity, transferase activity, glycosyltransferase activity
						TraesCS3B02G047300	Transmembrane transporter activity (transmembrane transport)
						TraesCS3B02G047600	ATP hydrolysis activity, ABC-type transporter activity (transmembrane transport)
STA_2	36892899_23_G/A	4A:619,145,783–619,605,783	6.73	< 0.01	265/273/270	TraesCS4A02G336800	Protein kinase activity (protein phosphorylation)
STA_3	10521380_21_A/G	4B:572,153,890–572,953,890	6.29	0.08	271/NA/256	NA	NA
STA_4	3606416_67_C/G	5B:488,820,374–489,660,374	11.95	0.35	279/244/260	TraesCS5B02G305100	Actin binding, actin filament binding (actin cytoskeleton organisation, actin nucleation)
						TraesCS5B02G305300 TraesCS5B02G305400 TraesCS5B02G305500	UDP-glycosyltransferase activity, hexosyltransferase activity
						TraesCS5B02G305600	UDP-glycosyltransferase activity
STA_5	3045349_41_T/G	5B:688,784,316–689,624,316	11.50	0.12	275/260/271	NA	NA
STA_6	22077643_9_T/G	NA	8.15	0.11	NA/236/272	NA	NA
STA_7	3043904_26_C/T	NA	9.60	0.31	265/272/276	NA	NA
STA_8	4350778_5_C/G	NA	6.18	0.18	272/243/236	NA	NA
**ADFom (g/kg DM)**							
ADFom_1	8536912_31_C/A	7A:704,030,351–704,630,351	7.10	0.03	260/235/250	TraesCS7A02G519800	Actin binding (regulation of actin filament polymerization, Arp2/3 complex-mediated actin nucleation)
ADFom_2	3610345_15_G/C	NA	15.08	0.38	243/NA/258	NA	NA
ADFom_3	54358455_11_T/C	NA	6.68	0.15	255/248/237	NA	NA
**Stem KL (g/kg aNDFom)**							
SKL_1	4366914_47_G/C	3A:685,868,646–687,208,646	8.25	0.13	129/129/126	TraesCS3A02G444500 TraesCS3A02G445900 TraesCS3A02G446800 TraesCS3A02G447300	Protein kinase activity, ATP binding (protein phosphorylation)
						TraesCS3A02G444600	Carbohydrate binding
						TraesCS3A02G446600 TraesCS3A02G446700	Potassium ion transmembrane transporter activity (potassium ion transmembrane transport)
SKL_2	4563722_14_A/G	7B:583,845,567–584,225,567	9.69	0.08	127/129/119	TraesCS7B02G328700	Transmembrane transporter activity (transmembrane transport)
						TraesCS7B02G328800	Protein kinase activity, ATP binding (protein phosphorylation)
SKL_3	4551138_8_C/G	NA	9.27	0.02	131/127/125	NA	NA
SKL_4	4212151_44_G/A	NA	6.85	0.07	128/131/127	NA	NA
SKL_5	15998020_11_A/C	NA	7.21	0.40	NA/117/130	NA	NA
SKL_6	3046493_23_G/A	NA	6.29	0.11	124/NA/132	NA	NA
SKL_7	4567564_20_T/G	NA	11.63	0.10	126/135/132	NA	NA
**Stem length (cm)**							
SL_1	8512008_13_G/C	NA	8.71	0.01	123/123/101	NA	NA
*SL_2*	*4370804_7_T/G*	*1R:675,629,057–676,729,057*	*8.06*	*0.04*	*100/147/113*	*NA*	*NA*
SL_3	4369682_17_C/G	NA	19.28	0.21	102/117/NA	NA	NA
SL_4	10519076_15_G/A	NA	8.34	0.10	NA/100/104	NA	NA
SL_5	15998020_11_A/C	NA	14.22	0.12	NA/91/107	NA	NA
SL_6	3621151_19_T/G	NA	10.45	0.07	107/105/93	NA	NA
SL_7	4208358_19_A/G	NA	19.58	0.21	100/127/144	NA	NA
SL_8	4348644_14_A/G	NA	6.19	0.02	102/104 /NA	NA	NA
SL_9	54357631_62_G/A	NA	20.33	0.15	150/NA/101	NA	NA
**Ear prop (%)**							
EP_1	4211801_19_C/T	1A:474,187,796–474,687,796	12.90	0.23	59.1/52.1/53.7	See QTL PIVNDFD_1	See QTL PIVNDFD_1
EP_2	8530946_28_G/A	5B:645,663,357–646,503,357	6.17	0.10	58.9/50.8/51.5	NA	NA
EP_3	10514469_23_C/G	NA	6.95	0.13	58.2/55.2/59.5	NA	NA
EP_4	36892695_26_C/G	NA	8.00	0.16	60.8/56.1/58.2	NA	NA
*EP_5*	*4209538_61_C/T*	*5R:610,604,285–611,704,285*	*11.36*	*0.27*	*59.9/56.6/56.7*	*NA*	*NA*

QTL: trait abbreviation followed by an ordinal number per trait; SNP marker: ID for the sequence in which the significant SNP marker occurs; Candidate region: chromosomal position of the significant SNP marker, expressed in basepairs (start and end position of the candidate region is defined by the LD decay distance around the significant SNP); P: - log10 of the P-value of the significant SNP marker; R²_p_: partial phenotypic variance explained by the significant SNP; Median BLUP value per genotypic class presented as 0/1/2 where 0: homozygous REF, 1: homozygous ALTERNATIVE, 2: heterozygous; Candidate gene: genes in the Wheat_ChineseSpring10 reference genome v1.0 (2018) and in the Secale cereale Lo7 v1 pseudomolecules (2021) reference genome in the candidate region defined by the LD decay distance of the different chromosomes around the significant SNP marker (retrieved from ‘The GrainGenes Genome Browsers’, https://wheat.pw.usda.gov/GG3/genome_browser); Annotation: function description retrieved from Ensembl Plants with the biological function in parenthesis (https://plants.ensembl.org/Triticum_aestivum/Info/Index, https://plants.ensembl.org/Secale_cereale/Info/Index). In italics: SNP markers with mapped positions to the rye reference genome after running a BLAST query (https://wheat.pw.usda.gov/blast/). NA: not available

A total of 258 genes of interest were identified within the candidate regions delineated around the significant SNP markers (Supplementary Table 3). A set of 55 of these genes is annotated with functions of particular relevance for plasma membrane transport, cytoskeleton organisation, enzyme activity, carbohydrate metabolic processes, protein phosphorylation, and sterol and CW biosynthesis (Table 3), which may influence IVOMD and IVNDFD. Furthermore, previously reported QTLs for important traits in wheat, including some related to FQ, are co-located with the candidate regions (Supplementary Table 3).

#### In vitro digestibility traits

Three SNP markers are significantly associated with plant IVOMD (Table 3) and explain individually 11 to 16% of the phenotypic variation (R²_p_). Candidate gene SECCE1Rv1G0007100 present in the candidate region surrounding SNP 10517787_19_C/T on chromosome 1R is putatively involved in plasma membrane transporter activity (Table 3). The candidate gene SECCE1Rv1G0007120 is annotated as a putative peroxidase (Table 3).

The highest number of significantly associated SNP markers was identified for plant IVNDFD. Ten SNP markers explain individually up to 27% of the phenotypic variation and are distributed over the three subgenomes (Table 3). The genes present in the candidate regions surrounding the significantly associated SNP markers are putatively involved in carbohydrate metabolic processes, plasma membrane transporter activity, exocytosis, and CW biosynthesis. The candidate region on chromosome 1A defined by SNP 4211801_19_C/T contains TraesCS1A02G278200, a gene annotated for carbohydrate and STA binding, and TraesCS1A02G279000, which plays a role in the carbohydrate metabolic process (Table 3). Furthermore, SNP 4211801_19_C/T co-locates with an interesting QTL for forage STA (Kukri_c3582_87, Supplementary Table 3). The candidate region on chromosome 2A defined by SNP 54358850_11_G/A contains TraesCS2A02G059800 which is related to transmembrane transport (Table 3). The candidate region surrounding SNP 54354903_15_A/G on chromosome 2B comprises candidate genes related to O-methyltransferase activity and UDP-glycosyltransferase activity (Table 3). Other candidate genes in this region have putative functions in transmembrane transport and exocytosis (Table 3). In addition, in the same region a QTL for plant height (WCSS1_contig5233676_2BS-10,473) is located (Supplementary Table 3). SNP 4370557_20_A/G on chromosome 5A co-locates with a QTL, CAP12_c1272_334, for peduncle length (Supplementary Table 3).

The eight SNP markers significantly associated with stem IVNDFD are distributed over the three subgenomes and explain 7 to 36% of the phenotypic variation (Table 3). The candidate region surrounding SNP 54345599_25_T/G on chromosome 2A contains TraesCS2A02G112200 which is involved in the glycosylphosphatidylinositol (GPI) anchor biosynthetic process (Table 3). The QTL WCSS1_contig5263571_2AS-613 for flag leaf stay-green period has also been reported for this region (Supplementary Table 3). The candidate region defined by SNP 4206397_9_C/A on chromosome 2R contains genes annotated with UDP-glycosyltransferase activity, transporter activity, and CW biogenesis (Table 3). More specifically, the candidate gene SECCE2Rv1G0126340 is involved in the xyloglucan metabolic process. In the candidate region on chromosome 3B, several QTLs were found for forage moisture, whole grain STA, flowering date, and spike weight (Supplementary Table 3).

#### Subtraits related to in vitro digestibility

For STA, eight SNP markers are identified as significant and explain up to 35% of the phenotypic variation (Table 3). The highest percentage of phenotypic variance is explained by SNP 3606416_67_C/G located on chromosome 5B (Table 3). Candidate genes in the candidate region surrounding SNP 11912016_38_T/C on chromosome 3B are involved in sterol biosynthesis, protein phosphorylation, and transmembrane transport (Table 3). Previously reported QTLs related to the number of grains/seeds and their characteristics, and forage protein co-locate with this chromosomal region (Supplementary Table 3). Gene TraesCS4A02G336800 present in the candidate region of SNP 36892899_23_G/A on chromosome 4A is also involved in protein phosphorylation (Table 3). QTLs for spike number have been reported in this region as well (Supplementary Table 3). In the present study, however, the QTL STA_2 explains very little of the variation in STA content (Table 3). SNP markers 3045349_41_T/G and 3606416_67_C/G are both located on chromosome 5B, with 199 Mbp between them (Table 3). Only for the latter SNP marker, genes of interest related to cytoskeleton organisation and UDP-glycosyltransferase activity were found (Table 3). Also in this case, QTLs previously reported for wheat co-locate with significant SNPs on chromosome 5B (Supplementary Table 3).

For ADFom, three SNP markers display significant associations, each explaining 3 to 38% of the phenotypic variation (Table 3). In contrast to SNP marker 8536912_31_C/A on chromosome 7A, the UM SNP markers explained most of the phenotypic variance (Table 3). The candidate region between 704,0 Mbp and 704,6 Mbp on chromosome 7A further contains TraesCS7A02G519800 annotated with actin binding which is of relevance for the cytoskeleton (Table 3) and a QTL for plant height (WCSS1_contig4557977_7AL-1122, Supplementary Table 3).

Seven SNP markers are found to display a significant association with stem KL. Their partial phenotypic variation ranges between 2 and 40% (Table 3). Several candidate genes are distributed over the candidate region surrounding SNP 4366914_47_G/C on chromosome 3A and are related to protein phosphorylation, carbohydrate binding, and transmembrane transport (Table 3). Many previously reported QTLs for flowering date, heading date, plant height, and kernel related traits are also present in this chromosomal region (Supplementary Table 3). In addition, two candidate genes in the candidate region of SNP 4563722_14_A/G on chromosome 7B are also relevant for protein phosphorylation and transmembrane transport (Table 3).

Although nine SNP markers are found significantly associated with stem length, no candidate genes are found related with this trait. The phenotypic variance explained by the significant SNP markers varied from 1 to 21% (Table 3). Only one marker was mapped to the rye reference genome (Table 3).

Lastly, significant SNP associations are detected for ear proportion on chromosomes 1A, 5B, and 5R (Table 3). Together with two UM markers, phenotypic variance explained by any individual SNP marker varied from 10 to 27% (Table 3). The candidate region of SNP 8530946_28_G/A on chromosome 5B contains one QTL for grain yield (WCSS1_contig10912959_5BL-4833, Supplementary Table 3).

## Discussion

### Triticale: a complex genome

Previous studies have already utilized markers based on DArT technology to investigate genetic diversity and loci that control agronomic traits in triticale [41–43, 61–63]. Despite the lack of a high-quality triticale reference genome, marker positions can be determined by mapping to the reference genomes of its progenitors, wheat and rye [64]. In this study, 57% of the 33,231 retained SNP DArTseq markers mapped to the A and B subgenomes of the wheat reference genome (Table 2), and several significantly trait-associated SNP markers could be mapped on the R subgenome using a BLAST query. Nevertheless, no chromosomal position could be determined on the wheat and rye genome for half of the significantly associated SNP markers that may be of interest for further investigations (Table 3). Due to sequence modifications and eliminations, large proportions of its parental genomes are not fully conserved in triticale [65, 66]. In this regard, approaches such as pan-genomics may better support the GWAS approach. Plant pan-genomes provide information on polymorphisms in different populations, where genetic variations can be genotyped relative to a pan-genome instead of a single reference genome [67].

For successful application of molecular markers in triticale breeding, the identification of tight MTAs is essential [4]. While this was already achieved for other traits, the use of GWAS to identify SNP markers of interest for IVD in triticale is demonstrated here. The identified MTAs were distributed across the A, B, and R subgenomes (Table 3), showing that the genetic basis for IVD and related subtraits in triticale is controlled by genomic regions in both the wheat and rye genome.

### Absence of major population structure in the studied winter triticale collection

Similarly to previous reports on genetic diversity in European winter triticale germplasm [62, 68, 69], no major population structuration was detected in the collection in this study (Fig. 2, PC1: 10.1% and PC2: 6.2%). The lack of population structure in European winter triticale can be explained by the extensive exchange of breeding material among Eastern Europe, Western Europe, North America, and Mexico (CIMMYT), what was stimulated after the development of superior Polish winter triticales in the 1980s [70, 71]. However, in agreement with Losert et al. [62], some clustering of genotypes of specific breeding origins was observed. Interestingly, the group containing six Romanian genotypes was genetically clearly differentiated from the other groups (Fig. 2). The incorporation of Russian material in addition to CIMMYT, Canadian, Polish, and German material possibly lays at the basis of the differentiation of the Romanian genotypes [72].

### Loci associated with in vitro digestibility and related traits

A GWAS is shown an effective approach for studying the genetic control of IVD and related subtraits in triticale, and more specifically in the winter triticale collection investigated here. It is known that the detection of associated SNP markers is more likely when large differences in phenotype are available [40]. Considerable variation and moderate to high H² for the investigated traits is shown in De Zutter et al. [44]. Despite the relatively small population size (118 genotypes) compared to previous GWAS in triticale [41–43, 73], 53 QTLs were identified over all investigated traits (Table 3). As expected for complex traits, most significantly associated markers were shown to explain only a small proportion of the phenotypic variation [74]. Regarding plant and stem IVNDFD, ten significant SNP markers were identified for plant IVNDFD explaining up to 27% of the phenotypic variance, compared with eight for stem IVNDFD explaining up to 36% (Table 3). This might reflect an even more complex genetic control of plant IVNDFD compared with stem IVNDFD. Moreover, IVNDFD of the triticale stem was found to be better predicted by NIRS compared to plant IVNDFD and both traits are well correlated (Spearman correlation coefficient: 0.79) [47]. Taken together, these results plead for the use of stem IVNDFD over plant IVNDFD for further breeding purposes.

Focusing on the stem, more specifically on a smaller stem length and a lower fibre fraction, is a proven approach to improve triticale FQ. Stem KL is already shown to be highly, negatively correlated with CWD in perennial ryegrass and maize stems [25, 26]. In wheat straw, Joshi et al. [39] detected that the markers Tdurum_contig76105_124 and Tdurum_contig76105_101 (624 mbp) on chromosome 3A were significantly associated with NDF, and marker wsnp_Ex_rep_c68058_66805898 (578 mbp) on chromosome 1A was significantly associated with ADF. An interesting marker for KL was found located on chromosome 3A (686 mbp) and one for IVNDFD on chromosome 1A (474 mbp) (Table 3), when considering the triticale stems. Unfortunately, the markers obtained are not closely located to the markers in the study of Joshi et al. [39] that is based on a panel of spring wheat lines which demonstrate a different development pattern compared to winter accessions.

In accordance with the genetic control of CW composition and digestibility in maize [34–36], triticale IVD and its related subtraits are mainly controlled by minor QTLs showing low to very low R²_p_ values. This is exemplified in the present study by the QTLs PIVNDFD_5, STA_2, and SL_1, which explain less than or 1% of variation (Table 3). Nevertheless, several significant SNP markers explained more than 30% of the phenotypic variance (Table 3), for example the STA SNP marker on chromosome 5B (3606416_67_C/G, R²_p_ = 35%) and an UM SNP for STA (3043904_26_C/T, R²_p_ = 31%). Given the high percentage of the phenotypic variance explained by this UM SNP, further investigations should focus on determining its location in the triticale genome.

Interestingly, one SNP marker provided evidence for a genetic relationship between several traits. SNP marker 4211801_19_C/T was associated with plant IVOMD, plant IVNDFD, and ear proportion (Fig. 4). A strong positive correlation (ρ = 0.79) was already observed between plant and stem IVNDFD at phenotypic level [44].The homozygous REF genotypic class of marker QTL 4211801_19_C/T displays higher plant and stem IVNDFD, suggesting that important loci controlling IVD in triticale are located on chromosome 1A (Fig. 4).

### Candidate genes for cell wall biosynthesis

Due to the lack of a high-quality triticale reference genome, wheat and rye reference genomes are useful resources for the identification of candidate genes that underlie markers associated with IVD traits in triticale [8]. Based on MTAs, candidate genes were identified which were of particular relevance for variation in IVD because they are putatively involved in plasma membrane transport, cytoskeleton organisation, enzyme activity, carbohydrate metabolic processes, protein phosphorylation, and sterol biogenesis (Table 3). More specifically, plasma membrane transport may be important to cross essential structural elements through the cell membrane for CW development [75, 76]. For example, ABC transporters, such as TraesCS2B02G041600 (Table 3), export complex molecular building blocks necessary for cell-type specific CW modifications and exocytosis provides components for CW architecture to the cell surface [77, 78]. In addition, it is known that the cytoskeleton regulates CW assembly [79, 80]. Peroxidases, such as SECCE1Rv1G0007120 (Table 3) may be involved in lignin cross-linking [75] and glycoside hydrolases, such as TraesCS1A02G279000 (Table 3), catalyse the hydrolysis of glycosidic bonds in complex sugars, including CELL and HCELL [81]. The GPI anchor biosynthetic process (Table 3) is crucial in CW metabolism and CW polymer cross-linking [82]. Moreover, the CELL content in CWs is found to be regulated by sterols and protein phosphorylation [83–85].

Furthermore, molecular markers should provide the opportunity to detect candidate genes involved in the CW biosynthesis of triticale as previously demonstrated in candidate-gene identification studies in maize [32–34]. Indeed, based on the significant association signals identified, candidate genes related to O-methyltransferase activity and UDP-glycosyltransferase activity (Table 3) are known to play a crucial role in the biosynthesis of lignin and CW polysaccharides, respectively [18, 75]. Interestingly, the xyloglucan-related candidate gene SECCE2Rv1G0126340 on chromosome 2R was found to be related to stem IVNDFD (Table 3). In the primary plant CW, xyloglucan is an essential building block for the biosynthesis of the CW polysaccharide HCELL, but its abundance is lower in grasses compared to dicots [86]. To date, it is still unclear whether xyloglucan decreases IVD, but it is associated with lignin, which is known to negatively affect CWD [16, 87]. Similarly, in elephant grass (*Cenchrus purpureus* (Schumach.) Morrone), a molecular marker associated with biomass digestibility was found in close proximity of the candidate gene Sevir.3G340800 (chromosome 3) which is involved in xyloglucan biosynthesis [88].

## Conclusions

It is essential to determine the genetic control of IVD to improve triticale forage quality by breeding. This study sheds light on the genetic architecture of IVD and its related subtraits in soft dough triticale forage using a GWAS. In accordance with previous studies in other forage crops, we demonstrated that improvement in IVD in triticale can be achieved by targeting individual plant organs. More specifically, reducing the KL content in the triticale stem offers great potential to improve the IVD of the total plant. At this stage, the genomic toolkit of triticale is still in its infancy. In the absence of a high-quality triticale reference genome, we had to rely on wheat and rye reference genomes. Nevertheless, interesting SNP markers on chromosomes 1A and 2R were identified that are significantly associated with stem CWD. To our knowledge, this is the first report that provide some genetic clues about CWD in triticale. These findings may be of particular interest for future marker-assisted selection (MAS) in triticale forage. In addition, more studies are required to further investigate the identified candidate genes and their usability in triticale. There are thus multiple opportunities for further research.

### Electronic supplementary material

Below is the link to the electronic supplementary material.

Supplementary Material 1

Supplementary Material 2

## Data Availability

All data generated or analysed during this study are included in this published article and its supplementary information files. The SNP marker data was kindly provided by Dr. Hans Peter Maurer of the Hohenheim University State Plant Breeding Institute, Stuttgart, Germany. The wheat and rye reference genome sequences in this article can be found from the GrainGenes Genome Browsers. The molecular function description of candidate genes and the biological processes in which they are putatively involved was retrieved from Ensembl Plants. The triticale seeds used in this study were provided by different breeding companies: Nordsaat Saatzucht GmbH (Germany), KWS Lochow (Germany), Danko Hodowla Roslin (Poland), SW Seed Lantmännen (the Netherlands), INCDA-Fundulea (Romania), HegeSaat GmbH & Co. KG (Germany), Semences Lemaire Deffontaines (France), DSP Delley (Switzerland), Florimond-Desprez (France), Northern Seed Montana (United States of America), Field Crop Development Centre at Lacombe (Canada), RAGT 2 n (France), Sejet Plant Breeding (Denmark), and Research Farm Bottelare (Belgium).

## Abbreviations

ADF: acid detergent fibre
ADFom: acid detergent fibre expressed exclusive of residual ash
ADL: acid detergent lignin
aNDFom: neutral detergent fibre assayed with a heat stable amylase and expressed exclusive of residual ash
BE: Belgium
BIC: Bayesian information criterion
BLINK: Bayesian-information and linkage-disequilibrium iteratively nested keyway
BLUP: best linear unbiased predictor
CA: Canada
CELL: cellulose
CH: Switzerland
Chr.: chromosome
CV: coefficient of variation
CW: cell wall
CWD: cell wall digestibility
DE: Germany
DK: Denmark
DM: dry matter
ear prop: ear proportion
FCDC: Field Crop Development Centre
FQ: forage quality
FR: France
GBS: genotyping-by-sequencing
GPI: glycosylphosphatidylinositol
GWAS: genome-wide association study
H²: broad-sense heritability
HCELL: hemicellulose
IVD: in vitro digestibility
IVNDFD: in vitro neutral detergent fibre digestibility
IVOMD: in vitro organic matter digestibility
KL: Klason lignin
LD: linkage disequilibrium
MAF: minor allele frequency
MAS: marker-assisted selection
MAX: maximum
MIN: minimum
MTA: marker-trait association
NDF: neutral detergent fibre
NIRS: near infrared spectroscopy
NL: the Netherlands
PC: principal component
PCA: principal components analysis
PL: Poland
Q-Q plot: Quantile-Quantile plot
QTL: quantitative trait locus
r²: square of the correlation coefficient
R²_p_: partial phenotypic variance explained by the significant SNP
RO: Romania
RPD: ratio of prediction to deviation
SD: standard deviation
SNP: single nucleotide polymorphism
STA: starch
UM: unmapped
US: United States
