# Supplementary material for: Genome-wide association study for in vitro digestibility and related traits in triticale forage
Source: BMC Plant Biol. 2024 Mar 27;24:223. doi: 10.1186/s12870-024-04927-7 (PMC10976741; doi:10.1186/s12870-024-04927-7)
Supplement: Supplementary file 1 — Supplementary Material 1 [file 12870_2024_4927_MOESM1_ESM.docx]

**Genome-wide association study for *in vitro* digestibility and related traits in triticale forage**

**BMC Plant Biology**

Anneleen De Zutter*, Maria Chiara Piro, Steven Maenhout, Hans Peter Maurer, Johan De Boever, Hilde Muylle, Isabel Roldán-Ruiz, and Geert Haesaert

* Corresponding author’s e-mail*:* [anneleen.dezutter@ugent.be](mailto:anneleen.dezutter@ugent.be)

Ghent University, Faculty of Bioscience Engineering, Department of Plants and Crops, Diepestraat 1, 9820 Bottelare, Belgium

**Supplemental material file**


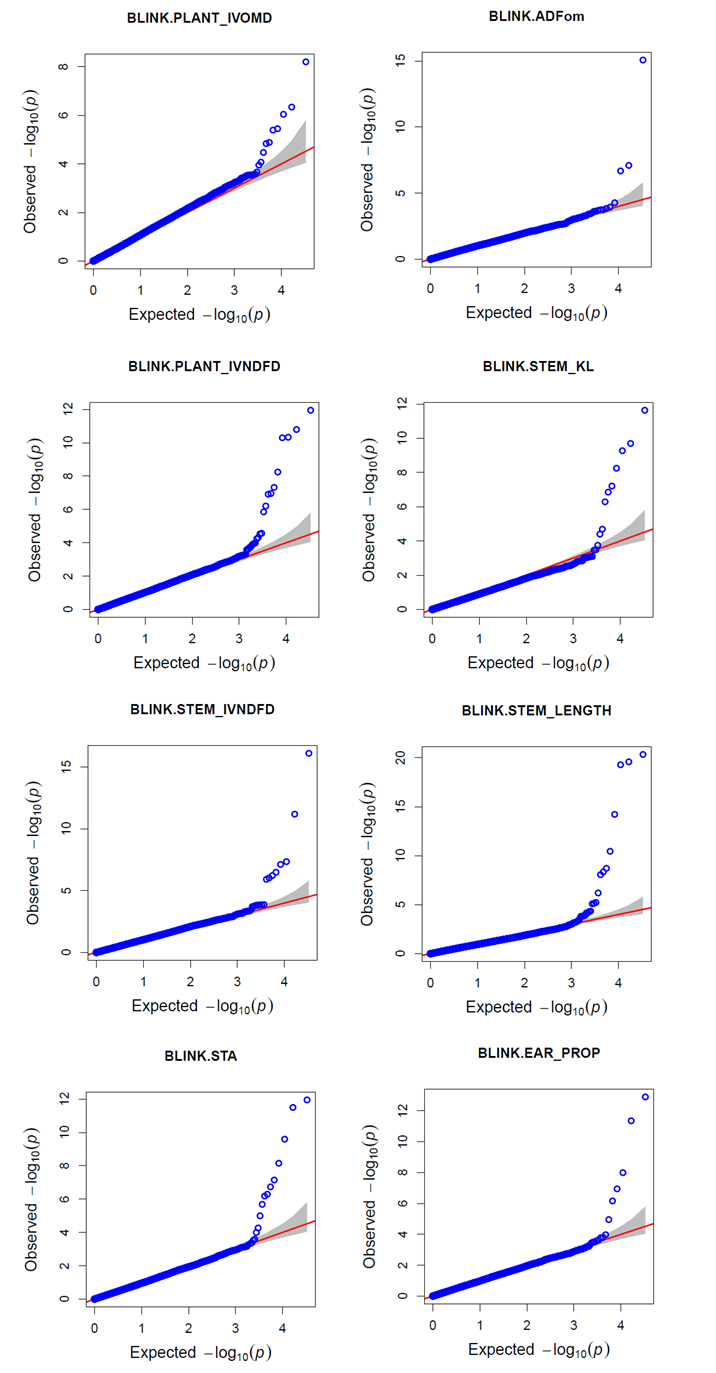


Supplementary Figure 1 Quantile-Quantile plots showing the relationship between the expected and obtained -log_10_ P-values from the GWAS for the different traits: total plant *in vitro* organic matter digestibility (plant_IVOMD), total plant *in vitro* neutral detergent fibre digestibility (plant_IVNDFD), stem *in vitro* neutral detergent fibre digestibility (stem_IVNDFD), total plant starch (STA), total plant acid detergent fibre expressed exclusive of residual ash (ADFom), stem Klason lignin (stem_KL), stem length, and ear proportion (ear_prop).

Supplementary Table 1 List of triticale varieties and breeding lines in the collection, arranged according to their country of breeding origin. Their year of release in shown in parentheses, except for the genotypes from the United States and the breeding lines. Sources: European plant variety catalogues & databases: https://ec.europa.eu/food/plant-variety-portal/ and [1].

| **Country of origin** | **Breeding company** | **Genotype name (year of release)** | |
| --- | --- | --- | --- |
| **Varieties** | | | |
| Canada | Field Crop Development Centre Lacombe | Bobcat (1999), Luoma (2008), Metzger (2009), Pika (1990) | 4 |
| Denmark | Sejet Plant Breeding | Jura (2013), Neogen (2016) | 2 |
| France | Florimond Desprez | Kereon (2010), Triade (2001), Tribeca (2007), Tricolor (1998), Trimaran (1992), Triskell (2005) | 6 |
|  | Lemaire Deffontaines Semences | Alambic (2012), Anagram (2015), Bienvenu (2001), Exagon (2013), Oxygen (2014) | 5 |
|  | RAGT Semences | Bellac (2000), Borodine (2007), Ragtac (2007), RGT Eleac (2016), RGT Expotrac (2016), RGT Flac (NA), RGT Omeac (2017), RGT Ruminac (2016), RGT Villarac (2016), Seconzac (2007), Tarzan (2009) | 11 |
| Germany | HegeSaat GmbH & Co. KG | Amarillo 105 (2007), Cortino (2012), Logo (2000), Massimo (2006), Trinidad (1996), Vuka (2009) | 6 |
|  | KWS Lochow GmbH | Cosinus (2009), KWS Aveo (2012), Mungis (2008), Rhenio (2014), Triamant (2003), Trimmer (2008) | 6 |
|  | Nordsaat Saatzucht GmbH | Claudius (2014), SU Agendus (2013), Tulus (2008) | 3 |
| Poland | Danko Hodowla Roslin | Atletico (2009), Benetto (2002), Dinaro (2004), Fidelio (1996), Fredro (2010), Kasyno (2016), Lamberto (1998), Lasko (1982), Madilo (2006), Magnat (2000), Moreno (1992), Mundo (1998), Orinoko (2017), Piano (1998), Porto (2017), Prego (1992), Salto (2015), Silverado (2013) | 18 |
| Romania | INCDA-Fundulea | Cascador F (2008), Haiduc (2009), Negoiu (2012), Pisc (2013), Stil (2009), Tulnic (2017) | 6 |
| Switzerland | Delley Seeds and Plants Ltd. | Bedretto (2003), Blenio (2002), Dorena (2007), Larossa (2014), Prader (2002), Timbo (2000), Trialdo (2011), Tridel (1997), Villars (2013) | 9 |
| The Netherlands | Lantmännen SW Seed B.V. | Adverdo (2013), Agostino (2009), Barolo (2015), Cedrico (2016), Cultivo (2007), Dometica (2016), Kaulos (2011), Kortego (2001), KWS Fido (2012), Lombardo (2015), Rotego (1998), SW Talentro (2002), Temuco (2016) | 13 |
| United States | Northern Seed Montana | 10T70126, TriCal 105, TriCal 115, TriCal 131, TriCal 135, TriCal 158, TriCal 336, TriCal 348, TriCal 813, TriCal 815, TriCal Flex 719, TriCal Gainer 154 | 12 |
| **Breeding lines** | | | |
| Belgium | Research Farm Bottelare | A12-02-430, A12-03-010, A12-03-033, A12-03-266, A12-08-290 | 5 |
| Canada | Field Crop Development Centre Lacombe | 00D016010, 00D016023, 02D006005, 02D006019, 03D005004, 06A049010, 06A051, 06D013002, 11B012, 11B019, 11B023, 11B025 | 12 |
|  |  | **Total:** | 118 |

Supplementary Table 2 Phenotypic traits evaluated in the triticale collection. Only those subtraits that were highly correlated with the *in vitro* digestibility (IVD) traits (Spearman correlation coefficient ρ > 0.50) [2] are considered in this study. In italics: plant height and ear length were used to calculate stem length.

| **Trait (unit)** | **Description** | **Method of determination** |
| --- | --- | --- |
| **IVD traits** | | |
| Plant IVOMD (%) | Total plant *in vitro* organic matter digestibility | Determined with NIRS from [3]. |
| Plant IVNDFD (%) | Total plant *in vitro* neutral detergent fibre digestibility | Determined with NIRS from [3]. |
| Stem IVNDFD (%) | Stem *in vitro* neutral detergent fibre digestibility | Determined with NIRS from [3]. |
| **IVD related subtraits** | | |
| STA (g/kg DM) | Total plant starch content | Determined with NIRS from [3]. |
| ADFom (g/kg DM) | Total plant acid detergent fibre content, expressed exclusive of residual ash | Determined with NIRS from [3]. |
| Stem KL (g/kg aNDFom) | Stem Klason lignin content of the main stem part between the second and third node, expressed on stem neutral detergent fibre content | Determined with NIRS from [3]. |
| Stem length (cm) | Length of the stem | Calculated as the difference between plant height and ear length on 6 main culms per microplot. Average value considered. |
| Ear prop (%) | Percentage of ear fraction on the total plant biomass | Calculated as fraction of ears on a total plant dry matter. |
| *Plant height (cm)* | *Total plant length from ground level, including ear but excluding awns* | *Determined on 6 main culms per microplot. Used to calculate stem length.* |
| *Ear length (cm)* | *Length of the ear, excluding awns* | *Determined on 6 main culms per microplot. Used to calculate stem length.* |

Supplementary Table 3 Results of the GWAS: significantly associated SNP markers with the studied traits (plant IVOMD, plant IVNDFD, stem IVNDFD, STA, ADFom, stem KL, stem length and ear proportion) and candidate genes in proximity of the significant SNP markers. QTL: trait abbreviation followed by an ordinal number per trait; SNP marker: ID for the sequence in which the significant SNP marker occurs; Candidate region: chromosomal position of the significant SNP marker, expressed in basepairs (start and end position of the candidate region is defined by the LD decay distance around the significant SNP); P: - log10 of the P-value of the significant SNP marker; R²p: partial phenotypic variance explained by the significant SNP; Median BLUP value per genotypic class presented as 0/1/2 where 0: homozygous REF, 1: homozygous ALTERNATIVE, 2: heterozygous; Known QTL: previously reported QTL in the wheat reference genome, obtained through the Triticeae Toolbox (T3) via ‘The GrainGenes Genome Browsers’ https://wheat.pw.usda.gov/GG3/genome_browser); Candidate gene: genes in the Wheat_ChineseSpring10 reference genome v1.0 (2018) and in the Secale cereale Lo7 v1 pseudomolecules (2021) reference genome in the candidate region defined by the LD decay distance of the different chromosomes around the significant SNP marker (retrieved from ‘The GrainGenes Genome Browsers’, https://wheat.pw.usda.gov/GG3/genome_browser); Annotation: function description retrieved from Ensembl Plants with the biological function in parenthesis (https://plants.ensembl.org/Triticum_aestivum/Info/Index, https://plants.ensembl.org/Secale_cereale/Info/Index). In italics: SNP markers with mapped positions to the rye reference genome after running a BLAST query (https://wheat.pw.usda.gov/blast/). NA: not available

| **QTL** | **SNP marker** | **Candidate region** | **P** | **R²_p_** | **Median BLUP value per genotypic class (0/1/2)** | **Known QTL** | **Candidate gene** | **Annotation (biological process)** | |
| --- | --- | --- | --- | --- | --- | --- | --- | --- | --- |
| **Plant IVOMD (%)** | | | | | | | | |  |
| *PIVOMD_1* | *10517787_19_C/T* | *1R:38333322-39433322* | *6.04* | *0.14* | *67.9/62.6/64.6* | *NA* | *SECCE1Rv1G0007060* | *UFM1 ligase activity*  *(protein ufmylation)* | |
|  |  |  |  |  |  |  | *SECCE1Rv1G0007070* | *UFM1 ligase activity*  *(protein ufmylation)* | |
|  |  |  |  |  |  |  | *SECCE1Rv1G0007080* | *(regulation of cilium movement)* | |
|  |  |  |  |  |  |  | *SECCE1Rv1G0007090* | *Kinase inhibitor activity* | |
|  |  |  |  |  |  |  | *SECCE1Rv1G0007100* | *Nucleotide binding, transporter activity, ATP binding, ATP hydrolysis activity* | |
|  |  |  |  |  |  |  | *SECCE1Rv1G0007120* | *Peroxidase activity, heme binding (response to oxidative stress, hydrogen peroxide catabolic process)* | |
|  |  |  |  |  |  |  | *SECCE1Rv1G0007140* | *NA* | |
|  |  |  |  |  |  |  | *SECCE1Rv1G0007170* | *Catalytic activity, methylthioadenosine nucleosidase activity*  *(nucleoside metabolic process, L-methionine salvage from methylthioadenosine)* | |
|  |  |  |  |  |  |  | *SECCE1Rv1G0007190* | *Potassium ion transmembrane transporter activity*  *(potassium ion transmembrane transport)* | |
| PIVOMD_2 | 22077643_9_T/G | NA | 8.20 | 0.16 | NA/62.1/67.7 |  | NA | NA | |
| PIVOMD_3 | 3042285_21_A/C | NA | 6.34 | 0.11 | 66.8/65.8/68.3 |  | NA | NA | |
| **Plant IVNDFD (%)** | | | | | | | | |  |
| PIVNDFD_1 | 4211801_19_C/T | 1A:474187796-474687796 | 6.96 | 0.05 | 55.1/50.4/50.5 | WCSS1_contig3886462_1AL-1148  (grain yield) | TraesCS1A02G278100 | Arginyltransferase activity  (protein arginylation) | |
|  |  |  |  |  |  | Kukri_c3582_87  (forage starch) | TraesCS1A02G278200 | Carbohydrate binding, starch binding | |
|  |  |  |  |  |  |  | TraesCS1A02G278300 | NA | |
|  |  |  |  |  |  |  | TraesCS1A02G278400 | NA | |
|  |  |  |  |  |  |  | TraesCS1A02G278500 | NA | |
|  |  |  |  |  |  |  | TraesCS1A02G278600 | Protein binding  (RNA processing) | |
|  |  |  |  |  |  |  | TraesCS1A02G278700 | Acyltransferase activity | |
|  |  |  |  |  |  |  | TraesCS1A02G278800 | NA | |
|  |  |  |  |  |  |  | TraesCS1A02G278900 | DNA binding | |
|  |  |  |  |  |  |  | TraesCS1A02G279000 | Hydrolase activity, hydrolyzing O-glycosyl compounds  (carbohydrate metabolic process) | |
| PIVNDFD_2 | 54358850_11_G/A | 2A:24803946-25923946 | 10.33 | 0.07 | 55.6/54.0/53.1 | WCSS1_contig5277131_2AS-1231  (grain hardness) | TraesCS2A02G058900 | (defense response) | |
|  |  |  |  |  |  | WCSS1_contig5277131_2AS-1349  (Li) | TraesCS2A02G059000 | NA | |
|  |  |  |  |  |  | WCSS1_contig5277131_2AS-1362  (harvest index) | TraesCS2A02G059200 | ADP binding  (defense response) | |
|  |  |  |  |  |  |  | TraesCS2A02G059300 | Protein binding | |
|  |  |  |  |  |  |  | TraesCS2A02G059400 | Monooxygenase activity, iron ion binding, oxidoreductase activity, acting on paired donors, with incorporation or reduction of molecular oxygen, heme binding | |
|  |  |  |  |  |  |  | TraesCS2A02G059500 | NA | |
|  |  |  |  |  |  |  | TraesCS2A02G059600 | NA | |
|  |  |  |  |  |  |  | TraesCS2A02G059700 | Protein binding | |
|  |  |  |  |  |  |  | TraesCS2A02G059800 | Cation:chloride symporter activity, transmembrane transporter activity  (ion transport, transmembrane transport) | |
| PIVNDFD_3 | 54354903_15_A/G | 2B:18789842-20039842 | 6.21 | 0.13 | 54.8/51.1/54.6 | Excalibur_c14396_1629  (flag leaf angle) | TraesCS2B02G041200 | Methyltransferase activity, O-methyltransferase activity, protein dimerization activity | |
|  |  |  |  |  |  | Excalibur_rep_c101263_892  (grain hardness) | TraesCS2B02G041300 | Acyltransferase activity, acyltransferase activity, transferring groups other than amino-acyl groups  (biosynthetic process) | |
|  |  |  |  |  |  | IWA7370  (stripe rust reaction type) | TraesCS2B02G041400 | UDP-glycosyltransferase activity | |
|  |  |  |  |  |  | WCSS1_contig5245446_2BS-9560  (Normalized Difference Vegetation Index) | TraesCS2B02G041500 | (plant-type sporogenesis, chromosome organization involved in meiotic cell cycle) | |
|  |  |  |  |  |  | gbsHWWAMP34933  (S) | TraesCS2B02G041600 | ATP binding, ATPase-coupled transmembrane transporter activity, ABC-type transporter activity  (transmembrane transport) | |
|  |  |  |  |  |  | gbsHWWAMP57558  (maturity date (physiological)) | TraesCS2B02G041700 | UDP-glycosyltransferase activity, oxidoreductase activity, acting on the CH-OH group of donors, NAD or NADP as acceptor, hexosyltransferase activity, NAD binding | |
|  |  |  |  |  |  | gbsHWWAMP11211  (spike color) | TraesCS2B02G041800 | UDP-glycosyltransferase activity, hexosyltransferase activity | |
|  |  |  |  |  |  | gbsHWWAMP11212  (Zn) | TraesCS2B02G041900 | (plant-type sporogenesis, chromosome organization involved in meiotic cell cycle) | |
|  |  |  |  |  |  | WCSS1_contig5233676_2BS-10473  (plant height) | TraesCS2B02G042000 | UDP-glycosyltransferase activity, hexosyltransferase activity | |
|  |  |  |  |  |  |  | TraesCS2B02G042100 | UDP-glycosyltransferase activity | |
|  |  |  |  |  |  |  | TraesCS2B02G042200 | UDP-glycosyltransferase activity, hexosyltransferase activity | |
|  |  |  |  |  |  |  | TraesCS2B02G042300 | (exocytosis, protein transport) | |
|  |  |  |  |  |  |  | TraesCS2B02G042400 | (exocytosis, protein transport) | |
|  |  |  |  |  |  |  | TraesCS2B02G042500 | ADP binding  (defense response) | |
|  |  |  |  |  |  |  | TraesCS2B02G042600 | ADP binding  (defense response) | |
|  |  |  |  |  |  |  | TraesCS2B02G042700 | ADP binding  (defense response) | |
|  |  |  |  |  |  |  | TraesCS2B02G042800 | ADP binding  (defense response) | |
|  |  |  |  |  |  |  | TraesCS2B02G042900 | (exocytosis, protein transport) | |
|  |  |  |  |  |  |  | TraesCS2B02G043000 | (exocytosis, protein transport) | |
|  |  |  |  |  |  |  | TraesCS2B02G043100 | (exocytosis, protein transport) | |
|  |  |  |  |  |  |  | TraesCS2B02G043200 | (exocytosis, protein transport) | |
| PIVNDFD_4 | 4370557_20_A/G | 5A:704492970-705452970 | 7.32 | 0.02 | 55.4/54.3/54.8 | BS00023138_51  (Li) | TraesCS5A02G551000 | NA | |
|  |  |  |  |  |  | CAP12_c1272_334  (peduncle length) | TraesCS5A02G551100 | NA | |
|  |  |  |  |  |  | WCSS1_contig2804867_5AL-10720  (grain weight) | TraesCS5A02G551200 | NA | |
|  |  |  |  |  |  | gbsHWWAMP10430  (Cd) | TraesCS5A02G551300 | NA | |
|  |  |  |  |  |  | gbsHWWAMP10431  (grain yield) | TraesCS5A02G551400 | NA | |
|  |  |  |  |  |  |  | TraesCS5A02G551500 | NA | |
|  |  |  |  |  |  |  | TraesCS5A02G551600 | NA | |
|  |  |  |  |  |  |  | TraesCS5A02G551700 | Protein binding | |
|  |  |  |  |  |  |  | TraesCS5A02G551800 | Protein binding | |
|  |  |  |  |  |  |  | TraesCS5A02G551900 | NA | |
|  |  |  |  |  |  |  | TraesCS5A02G552000 | Nicotianamine synthase activity  (nicotianamine biosynthetic process) | |
|  |  |  |  |  |  |  | TraesCS5A02G552100 | NA | |
|  |  |  |  |  |  |  | TraesCS5A02G552200 | Protein kinase activity, ATP binding  (protein phosphorylation) | |
|  |  |  |  |  |  |  | TraesCS5A02G552300 | rRNA N-glycosylase activity, toxin activity  (defense response,  negative regulation of translation) | |
|  |  |  |  |  |  |  | TraesCS5A02G552400 | Transferase activity,  nicotianamine synthase activity  (nicotianamine biosynthetic process) | |
|  |  |  |  |  |  |  | TraesCS5A02G552500 | rRNA N-glycosylase activity  (negative regulation of translation) | |
|  |  |  |  |  |  |  | TraesCS5A02G552600 | Actin binding | |
|  |  |  |  |  |  |  | TraesCS5A02G552700 | (mRNA export from nucleus, ethylene-activated signaling pathway, primary ta-siRNA processing, defense response to fungus) | |
| PIVNDFD_5 | 3623312_68_T/C | NA | 5.85 | 0.01 | 54.2/52.8/55.9 |  | NA | NA | |
| PIVNDFD_6 | 4567261_61_T/C | 7A:284227044-284827044 | 8.24 | 0.10 | NA/54.8/54.1 | BobWhite_c20374_162  (canopy temperature depression (grain fill)) | TraesCS7A02G271200 | Monooxygenase activity, iron ion binding, oxidoreductase activity, acting on paired donors, with incorporation or reduction of molecular oxygen,  heme binding | |
|  |  |  |  |  |  | Kukri_c55196_445  (canopy temperature depression (grain fill)) | TraesCS7A02G271300 | NA | |
|  |  |  |  |  |  |  | TraesCS7A02G271400 | ER retention sequence binding  (protein retention in ER lumen, protein transport) | |
|  |  |  |  |  |  |  | TraesCS7A02G271500 | NA | |
|  |  |  |  |  |  |  | TraesCS7A02G271600 | NA | |
| PIVNDFD_7 | 14469989_24_A/G | NA | 6.91 | 0.11 | 54.9/NA/49.2 |  | NA | NA | |
| PIVNDFD_8 | 15998020_11_A/C | NA | 11.95 | 0.27 | NA/57.3/54.1 |  | NA | NA | |
| PIVNDFD_9 | 22077643_9_T/G | NA | 10.80 | 0.18 | NA/49.5/54.9 |  | NA | NA | |
| *PIVNDFD_10* | *3046586_39_G/A* | *5R:609761318-610861318* | *10.30* | *0.20* | *55.6/50.5/53.7* |  | *SECCE5Rv1G0339690* | *NA* | |
|  |  |  |  |  |  |  | *SECCE5Rv1G0339700* | *NA* | |
|  |  |  |  |  |  |  | *SECCE5Rv1G0339710* | *NA* | |
|  |  |  |  |  |  |  | *SECCE5Rv1G0339720* | *DNA-binding transcription factor activity, protein dimerization activity*  *(regulation of transcription, DNA-templated)* | |
|  |  |  |  |  |  |  | *SECCE5Rv1G0339730* | *DNA-binding transcription factor activity, protein dimerization activity*  *(regulation of transcription, DNA-templated)* | |
|  |  |  |  |  |  |  | *SECCE5Rv1G0339740* | *DNA-binding transcription factor activity, protein dimerization activity*  *(regulation of transcription, DNA-templated)* | |
|  |  |  |  |  |  |  | *SECCE5Rv1G0339760* | *DNA-binding transcription factor activity*  *(regulation of transcription, DNA-templated)* | |
|  |  |  |  |  |  |  | *SECCE5Rv1G0339770* | *DNA-binding transcription factor activity, protein dimerization activity*  *(regulation of transcription, DNA-templated)* | |
| **Stem IVNDFD (%)** | | | | | | | | |  |
| SIVNDFD_1 | 4211801_19_C/T | 1A:474187796-474687796 | 16.12 | 0.14 | 38.0/32.1/33.3 |  | See QTL PIVNDFD_1 | See QTL PIVNDFD_1 | |
| SIVNDFD_2 | 54345599_25_T/G | 2A:62591738-63711738 | 6.04 | 0.10 | 37.4/37.6/NA | WCSS1_contig5231985_2AS-526  (test weight) | TraesCS2A02G111400 | NA | |
|  |  |  |  |  |  | WCSS1_contig5231985_2AS-493  (test weight) | TraesCS2A02G111500 | NA | |
|  |  |  |  |  |  | WCSS1_contig5263571_2AS-613  (flag leaf stay-green period) | TraesCS2A02G111600 | Nucleic acid binding, RNA binding | |
|  |  |  |  |  |  |  | TraesCS2A02G111700 | NA | |
|  |  |  |  |  |  |  | TraesCS2A02G111800 | Protein binding | |
|  |  |  |  |  |  |  | TraesCS2A02G111900 | Protein binding | |
|  |  |  |  |  |  |  | TraesCS2A02G112000 | Protein binding | |
|  |  |  |  |  |  |  | TraesCS2A02G112100 | NA | |
|  |  |  |  |  |  |  | TraesCS2A02G112200 | Acyltransferase activity  (GPI anchor biosynthetic process) | |
|  |  |  |  |  |  |  | TraesCS2A02G112300 | GTP binding | |
|  |  |  |  |  |  |  | TraesCS2A02G112400 | NA | |
|  |  |  |  |  |  |  | TraesCS2A02G112500 | NA | |
|  |  |  |  |  |  |  | TraesCS2A02G112600 | NA | |
|  |  |  |  |  |  |  | TraesCS2A02G112700 | RNA binding  (tRNA modification) | |
| *SIVNDFD_3* | *4206397_9_C/A* | *2R:844945966-846045966* | *6.49* | *0.10* | *37.2/39.0/39.6* |  | *SECCE2Rv1G0126180* | *(defense response to other organism)* | |
|  |  |  |  |  |  |  | *SECCE2Rv1G0126190* | *NA* | |
|  |  |  |  |  |  |  | *SECCE2Rv1G0126200* | *UDP-glycosyltransferase activity* | |
|  |  |  |  |  |  |  | *SECCE2Rv1G0126210* | *NA* | |
|  |  |  |  |  |  |  | *SECCE2Rv1G0126220* | *ATP binding, ABC-type transporter activity* | |
|  |  |  |  |  |  |  | *SECCE2Rv1G0126240* | *Zinc ion binding* | |
|  |  |  |  |  |  |  | *SECCE2Rv1G0126260* | *Transmembrane transporter activity*  *(transmembrane transport)* | |
|  |  |  |  |  |  |  | *SECCE2Rv1G0126280* | *Transmembrane transporter activity*  *(transmembrane transport)* | |
|  |  |  |  |  |  |  | *SECCE2Rv1G0126290* | *Transmembrane transporter activity*  *(transmembrane transport)* | |
|  |  |  |  |  |  |  | *SECCE2Rv1G0126300* | *Transmembrane transporter activity*  *(transmembrane transport)* | |
|  |  |  |  |  |  |  | *SECCE2Rv1G0126310* | *Transmembrane transporter activity*  *(transmembrane transport)* | |
|  |  |  |  |  |  |  | *SECCE2Rv1G0126320* | *Transmembrane transporter activity*  *(transmembrane transport)* | |
|  |  |  |  |  |  |  | *SECCE2Rv1G0126330* | *NA* | |
|  |  |  |  |  |  |  | *SECCE2Rv1G0126340* | *Hydrolase activity, hydrolyzing O-glycosyl compounds,*  *xyloglucan:xyloglucosyl transferase activity*  *(carbohydrate metabolic process, cellular glucan metabolic process, xyloglucan metabolic process, cell wall biogenesis)* | |
|  |  |  |  |  |  |  | *SECCE2Rv1G0126360* | *Oligopeptide transmembrane transporter activity* | |
|  |  |  |  |  |  |  | *SECCE2Rv1G0126370* | *Oligopeptide transmembrane transporter activity* | |
| SIVNDFD_4 | 10514146_31_A/C | 3B:763613048-764533048 | 7.13 | 0.11 | 37.2/35.8/37.8 | WCSS1_contig9558833_3B-121  (whole grain starch) | TraesCS3B02G520700 | Catalytic activity | |
|  |  |  |  |  |  | WCSS1_contig9558833_3B-87  (whole grain starch) | TraesCS3B02G520794 | Protein binding | |
|  |  |  |  |  |  | WCSS1_contig10552932_3B-640  (forage moisture) | TraesCS3B02G520799 | NA | |
|  |  |  |  |  |  | WCSS1_contig10474029_3B-4249  (whole grain starch) | TraesCS3B02G520900 | Protein binding | |
|  |  |  |  |  |  | WCSS1_contig10766585_3B-7657  (Mg) | TraesCS3B02G521000 | Protein binding | |
|  |  |  |  |  |  | WCSS1_contig10766585_3B-7667  (Cu) | TraesCS3B02G521200 | NA | |
|  |  |  |  |  |  | WCSS1_contig10766585_3B-8480  (grain yield) |  |  | |
|  |  |  |  |  |  | WCSS1_contig10766585_3B-8481  (grain yield) |  |  | |
|  |  |  |  |  |  | WCSS1_contig10775754_3B-8498  (flowering date) |  |  | |
|  |  |  |  |  |  | gbsHWWAMP2899  (spike weight) |  |  | |
|  |  |  |  |  |  | WCSS1_contig10766867_3B-2921  (spike weight) |  |  | |
| SIVNDFD_5 | 3607057_38_A/G | NA | 7.35 | 0.10 | NA/41.9/36.6 |  | NA | NA | |
| SIVNDFD_6 | 54360481_5_G/T | NA | 6.22 | 0.07 | NA/35.9/39.7 |  | NA | NA | |
| SIVNDFD_7 | 15998020_11_A/C | NA | 11.20 | 0.36 | NA/43.2/37.0 |  | NA | NA | |
| SIVNDFD_8 | 8539528_31_A/G | NA | 5.92 | 0.15 | NA/35.1/37.9 |  | NA | NA | |
| **STA (g/kg DM)** | | | | | | | | |  |
| STA_1 | 11912016_38_T/C | 3B:23305225-24225225 | 7.14 | 0.12 | 270/259/272 | Kukri_c13830_924  (seeds per head) | TraesCS3B02G046000 | Nucleic acid binding | |
|  |  |  |  |  |  | RFL_Contig5043_785  (seeds per head) | TraesCS3B02G046100 | C-5 sterol desaturase activity, iron ion binding, octadecanal decarbonylase activity, oxidoreductase activity, lyase activity, aldehyde oxygenase (deformylating) activity  (response to stress, lipid biosynthetic process, sterol biosynthetic process,  response to oxygen-containing compound) | |
|  |  |  |  |  |  | gbsHWWAMP7330  (grain fill duration) | TraesCS3B02G046200 | Protein binding | |
|  |  |  |  |  |  | gbsHWWAMP7331  (seeds per head) | TraesCS3B02G046300 | Protein binding | |
|  |  |  |  |  |  | gbsHWWAMP7332  (thousand kernel weight) | TraesCS3B02G046400 | Protein binding | |
|  |  |  |  |  |  | WCSS1_contig10405673_3B-3788  (grain width) | TraesCS3B02G046500 | (peptidyl-lysine modification to peptidyl-hypusine) | |
|  |  |  |  |  |  | WCSS1_contig10405673_3B-3809  (grain width) | TraesCS3B02G046600 | Protein binding | |
|  |  |  |  |  |  | Tdurum_contig43252_1762  (test weight) | TraesCS3B02G046700 | Protein kinase activity, ATP binding  (protein phosphorylation) | |
|  |  |  |  |  |  | Tdurum_contig43252_1407  (grain yield) | TraesCS3B02G046800 | RNA binding, translation initiation factor activity  (translational initiation) | |
|  |  |  |  |  |  | BS00044752_51  (forage protein) | TraesCS3B02G046900 | Acetylglucosaminyltransferase activity, transferase activity, glycosyltransferase activity | |
|  |  |  |  |  |  | Tdurum_contig93037_960  (Normalized water index 3) | TraesCS3B02G047000 | Acetylglucosaminyltransferase activity, transferase activity, glycosyltransferase activity | |
|  |  |  |  |  |  |  | TraesCS3B02G047100 | NA | |
|  |  |  |  |  |  |  | TraesCS3B02G047200 | (regulation of transcription, DNA-templated) | |
|  |  |  |  |  |  |  | TraesCS3B02G047300 | Transmembrane transporter activity  (transmembrane transport) | |
|  |  |  |  |  |  |  | TraesCS3B02G047400 | NA | |
|  |  |  |  |  |  |  | TraesCS3B02G047500 | Nucleotide binding, DNA helicase activity, helicase activity, ATP binding, ATP-dependent activity, acting on DNA, hydrolase activity, 5'-3' DNA helicase activity  (box C/D snoRNP assembly, chromatin remodelling, regulation of transcription by RNA polymerase II, histone acetylation, DNA duplex unwinding) | |
|  |  |  |  |  |  |  | TraesCS3B02G047600 | Nucleotide binding, ATP binding, ATP hydrolysis activity, ABC-type transporter activity  (transmembrane transport) | |
|  |  |  |  |  |  |  | TraesCS3B02G047700 | NA | |
|  |  |  |  |  |  |  | TraesCS3B02G047800 | NA | |
| STA_2 | 36892899_23_G/A | 4A:619145783-619605783 | 6.73 | <0.01 | 265/273/270 | gbsHWWAMP54609  (spike number) | TraesCS4A02G336600 | NA | |
|  |  |  |  |  |  | Excalibur_rep_c104791_231  (S) | TraesCS4A02G336700 | DNA binding | |
|  |  |  |  |  |  | WCSS1_contig7133560_4AL-4666  (spike number) | TraesCS4A02G336800 | Protein kinase activity, ATP binding  (protein phosphorylation) | |
|  |  |  |  |  |  |  | TraesCS4A02G336900 | NA | |
|  |  |  |  |  |  |  | TraesCS4A02G337000 | NA | |
|  |  |  |  |  |  |  | TraesCS4A02G337100 | NA | |
|  |  |  |  |  |  |  | TraesCS4A02G337200 | NA | |
|  |  |  |  |  |  |  | TraesCS4A02G337300 | Catalytic activity | |
|  |  |  |  |  |  |  | TraesCS4A02G337400 | NA | |
|  |  |  |  |  |  |  | TraesCS4A02G337500 | NA | |
| STA_3 | 10521380_21_A/G | 4B:572153890-572953890 | 6.29 | 0.08 | 271/NA/256 |  | TraesCS4B02G287900 | Protein dimerization activity  (regulation of transcription, DNA-templated, regulation of growth, regulation of seed growth) | |
| STA_4 | 3606416_67_C/G | 5B:488820374-489660374 | 11.95 | 0.35 | 279/244/260 | Jagger_c3991_101  (heading date) | TraesCS5B02G304800 | NA | |
|  |  |  |  |  |  | gbsCNLmaster31876  (heading date) | TraesCS5B02G304900 | ADP binding  (defense response) | |
|  |  |  |  |  |  | GENE-3437_68  (heading date) | TraesCS5B02G305000 | NA | |
|  |  |  |  |  |  | GENE-3437_148  (grain yield) | TraesCS5B02G305100 | Actin binding, actin filament binding  (actin cytoskeleton organization, actin nucleation) | |
|  |  |  |  |  |  |  | TraesCS5B02G305200 | NA | |
|  |  |  |  |  |  |  | TraesCS5B02G305300 | UDP-glycosyltransferase activity, hexosyltransferase activity | |
|  |  |  |  |  |  |  | TraesCS5B02G305400 | UDP-glycosyltransferase activity, hexosyltransferase activity | |
|  |  |  |  |  |  |  | TraesCS5B02G305500 | UDP-glycosyltransferase activity, hexosyltransferase activity | |
|  |  |  |  |  |  |  | TraesCS5B02G305600 | UDP-glycosyltransferase activity | |
|  |  |  |  |  |  |  | TraesCS5B02G305700 | Protein binding | |
| STA_5 | 3045349_41_T/G | 5B:688784316-689624316 | 11.50 | 0.12 | 275/260/271 | WCSS1_contig10918844_5BL-6091  (plant height) | TraesCS5B02G530000 | Aspartic-type endopeptidase activity | |
|  |  |  |  |  |  | WCSS1_contig10918844_5BL-6074  (Co) | TraesCS5B02G530100 | Guanyl-nucleotide exchange factor activity  (vesicle-mediated transport) | |
|  |  |  |  |  |  | gbsCNLmaster44704  (plant height) | TraesCS5B02G530200 | NA | |
|  |  |  |  |  |  | gbsHWWAMP24965  (thousand kernel weight) | TraesCS5B02G530300 | Guanyl-nucleotide exchange factor activity  (vesicle-mediated transport) | |
|  |  |  |  |  |  | Excalibur_c54637_123  (seeds per head) | TraesCS5B02G530400 | NA | |
|  |  |  |  |  |  |  | TraesCS5B02G530500 | Oxidoreductase activity | |
|  |  |  |  |  |  |  | TraesCS5B02G530600 | NA | |
|  |  |  |  |  |  |  | TraesCS5B02G530700 | Protein binding | |
|  |  |  |  |  |  |  | TraesCS5B02G530800 | GTPase activity, GTP binding | |
|  |  |  |  |  |  |  | TraesCS5B02G530900 | N,N-dimethylaniline monooxygenase activity, flavin adenine dinucleotide binding, NADP binding | |
|  |  |  |  |  |  |  | TraesCS5B02G531000 | N,N-dimethylaniline monooxygenase activity, flavin adenine dinucleotide binding, NADP binding | |
|  |  |  |  |  |  |  | TraesCS5B02G531100 | (lipid transport) | |
|  |  |  |  |  |  |  | TraesCS5B02G531200 | (lipid transport) | |
|  |  |  |  |  |  |  | TraesCS5B02G531300 | NA | |
|  |  |  |  |  |  |  | TraesCS5B02G531400 | (response to desiccation, response to cytokinin, response to cadmium ion) | |
|  |  |  |  |  |  |  | TraesCS5B02G531500 | NA | |
| STA_6 | 22077643_9_T/G | NA | 8.15 | 0.11 | NA/236/272 |  | NA | NA | |
| STA_7 | 3043904_26_C/T | NA | 9.60 | 0.31 | 265/272/276 |  | NA | NA | |
| STA_8 | 4350778_5_C/G | NA | 6.18 | 0.18 | 272/243/236 |  | NA | NA | |
| **ADFom (g/kg DM)** | | | | | | | | |  |
| ADFom_1 | 8536912_31_C/A | 7A:704030351-704630351 | 7.10 | 0.03 | 260/235/250 | WCSS1_contig4557977_7AL-1122  (plant height) | TraesCS7A02G519400 | NA | |
|  |  |  |  |  |  |  | TraesCS7A02G519500 | NA | |
|  |  |  |  |  |  |  | TraesCS7A02G519600 | RNA binding, structural constituent of ribosome  (translation) | |
|  |  |  |  |  |  |  | TraesCS7A02G519700 | DNA binding, DNA-directed 5'-3' RNA polymerase activity, protein dimerization activity  (transcription, DNA-templated) | |
|  |  |  |  |  |  |  | TraesCS7A02G519800 | Actin binding  (regulation of actin filament polymerization, Arp2/3 complex-mediated actin nucleation) | |
|  |  |  |  |  |  |  | TraesCS7A02G519900 | Protein binding | |
|  |  |  |  |  |  |  | TraesCS7A02G520000 | (defense response) | |
|  |  |  |  |  |  |  | TraesCS7A02G520100 | NA | |
|  |  |  |  |  |  |  | TraesCS7A02G520300 | (vacuolar transport) | |
| ADFom_2 | 3610345_15_G/C | NA | 15.08 | 0.38 | 243/NA/258 |  | NA | NA | |
| ADFom_3 | 54358455_11_T/C | NA | 6.68 | 0.15 | 255/248/237 |  | NA | NA | |
| **Stem KL (g/kg aNDFom)** | | | | | | | | |  |
| SKL_1 | 4366914_47_G/C | 3A:685868646-687208646 | 8.25 | 0.13 | 129/129/126 | Ex_c24554_1583  (flowering date) | TraesCS3A02G444300 | Oxidoreductase activity, oxidoreductase activity, acting on the CH-OH group of donors, NAD or NADP as acceptor | |
|  |  |  |  |  |  | Excalibur_c20392_1075  (plant height) | TraesCS3A02G444400 | NA | |
|  |  |  |  |  |  | BobWhite_c38444_238  (spike number) | TraesCS3A02G444500 | Protein kinase activity, protein binding, ATP binding  (protein phosphorylation) | |
|  |  |  |  |  |  | gbsHWWAMP46383  (Co) | TraesCS3A02G444600 | Carbohydrate binding | |
|  |  |  |  |  |  | BS00060029_51  (thousand kernel weight) | TraesCS3A02G444700 | NA | |
|  |  |  |  |  |  | TA003342-0357  (canopy senescence score T7) | TraesCS3A02G444800 | NA | |
|  |  |  |  |  |  | RAC875_c4641_773  (test weight) | TraesCS3A02G444900 | NA | |
|  |  |  |  |  |  | Excalibur_c54205_336  (canopy senescence score T7) | TraesCS3A02G445000 | Oxidoreductase activity, metal ion binding | |
|  |  |  |  |  |  | Kukri_rep_c115341_118  (Fe) | TraesCS3A02G445100 | Protein binding | |
|  |  |  |  |  |  | Excalibur_c13154_134  (heading date) | TraesCS3A02G445200 | Protein binding | |
|  |  |  |  |  |  | Tdurum_contig68305_597  (heading date) | TraesCS3A02G445300 | NA | |
|  |  |  |  |  |  | BS00088756_51  (plant height) | TraesCS3A02G445400 | NA | |
|  |  |  |  |  |  | Tdurum_contig68305_703  (heading date) | TraesCS3A02G445500 | NA | |
|  |  |  |  |  |  | Tdurum_contig68305_796  (plant height) | TraesCS3A02G445600 | NA | |
|  |  |  |  |  |  | IWA1551  (plant height) | TraesCS3A02G445700 | Protein binding | |
|  |  |  |  |  |  | RAC875_rep_c75557_177  (plant height) | TraesCS3A02G445800 | Protein binding | |
|  |  |  |  |  |  | WCSS1_contig4319573_3AL-2930  (grain fill duration) | TraesCS3A02G445900 | Protein kinase activity, protein binding, ATP binding  (protein phosphorylation) | |
|  |  |  |  |  |  | gbsHWWAMP34258  (single kernel weight) | TraesCS3A02G446000 | Protein binding | |
|  |  |  |  |  |  | BobWhite_c5337_225  (plant height) | TraesCS3A02G446100 | Protein binding | |
|  |  |  |  |  |  | WCSS1_contig4414496_3AL-1934  (spike weight) | TraesCS3A02G446200 | Serine-type carboxypeptidase activity  (proteolysis) | |
|  |  |  |  |  |  |  | TraesCS3A02G446300 | RNA binding, protein binding | |
|  |  |  |  |  |  |  | TraesCS3A02G446400 | Protein binding | |
|  |  |  |  |  |  |  | TraesCS3A02G446500 | NA | |
|  |  |  |  |  |  |  | TraesCS3A02G446600 | Potassium ion transmembrane transporter activity  (potassium ion transmembrane transport) | |
|  |  |  |  |  |  |  | TraesCS3A02G446700 | Potassium ion transmembrane transporter activity  (potassium ion transmembrane transport) | |
|  |  |  |  |  |  |  | TraesCS3A02G446800 | Protein kinase activity, ATP binding  (protein phosphorylation, signal transduction) | |
|  |  |  |  |  |  |  | TraesCS3A02G446900 | NA | |
|  |  |  |  |  |  |  | TraesCS3A02G447000 | NA | |
|  |  |  |  |  |  |  | TraesCS3A02G447100 | Enzyme inhibitor activity | |
|  |  |  |  |  |  |  | TraesCS3A02G447200 | Enzyme inhibitor activity | |
|  |  |  |  |  |  |  | TraesCS3A02G447300 | Protein kinase activity, ATP binding  (protein phosphorylation) | |
| SKL_2 | 4563722_14_A/G | 7B:583845567-584225567 | 9.69 | 0.08 | 127/129/119 |  | TraesCS7B02G328700 | Nitrate transmembrane transporter activity, transmembrane transporter activity  (transmembrane transport) | |
|  |  |  |  |  |  |  | TraesCS7B02G328800 | Protein kinase activity, ATP binding  (protein phosphorylation, recognition of pollen) | |
|  |  |  |  |  |  |  | TraesCS7B02G328900 | NA | |
|  |  |  |  |  |  |  | TraesCS7B02G329000 | Hydrolase activity, acting on ester bonds | |
|  |  |  |  |  |  |  | TraesCS7B02G329100 | NA | |
| SKL_3 | 4551138_8_C/G | NA | 9.27 | 0.02 | 131/127/125 |  | NA | NA | |
| SKL_4 | 4212151_44_G/A | NA | 6.85 | 0.07 | 128/131/127 |  | NA | NA | |
| SKL_5 | 15998020_11_A/C | NA | 7.21 | 0.40 | NA/117/130 |  | NA | NA | |
| SKL_6 | 3046493_23_G/A | NA | 6.29 | 0.11 | 124/NA/132 |  | NA | NA | |
| SKL_7 | 4567564_20_T/G | NA | 11.63 | 0.10 | 126/135/132 |  | NA | NA | |
| **Stem length (cm)** | | | | | | | | |  |
| SL_1 | 8512008_13_G/C | NA | 8.71 | 0.01 | 123/123/101 |  | NA | NA | |
| *SL_2* | *4370804_7_T/G* | *1R:675629057-676729057* | *8.06* | *0.04* | *100/147/113* |  | *SECCE1Rv1G0054660* | *NA* | |
|  |  |  |  |  |  |  | *SECCE1Rv1G0054720* | *Protein binding* | |
|  |  |  |  |  |  |  | *SECCE1Rv1G0054730* | *Hydrolase activity, acting on ester bonds* | |
|  |  |  |  |  |  |  | *SECCE1Rv1G0054760* | *NA* | |
|  |  |  |  |  |  |  | *SECCE1Rv1G0054830* | *Protein binding* | |
|  |  |  |  |  |  |  | *SECCE1Rv1G0054850* | *Protein binding* | |
|  |  |  |  |  |  |  | *SECCE1Rv1G0054860* | *Asparagine synthase (glutamine-hydrolyzing) activity*  *(asparagine biosynthetic process)* | |
|  |  |  |  |  |  |  | *SECCE1Rv1G0054870* | *Protein phosphatase regulator activity*  *(signal transduction)* | |
| SL_3 | 4369682_17_C/G | NA | 19.28 | 0.21 | 102/117/NA |  | NA | NA | |
| SL_4 | 10519076_15_G/A | NA | 8.34 | 0.10 | NA/100/104 |  | NA | NA | |
| SL_5 | 15998020_11_A/C | NA | 14.22 | 0.12 | NA/91/107 |  | NA | NA | |
| SL_6 | 3621151_19_T/G | NA | 10.45 | 0.07 | 107/105/93 |  | NA | NA | |
| SL_7 | 4208358_19_A/G | NA | 19.58 | 0.21 | 100/127/144 |  | NA | NA | |
| SL_8 | 4348644_14_A/G | NA | 6.19 | 0.02 | 102/104 /NA |  | NA | NA | |
| SL_9 | 54357631_62_G/A | NA | 20.33 | 0.15 | 150/NA/101 |  | NA | NA | |
| **Ear prop (%)** | | | | | | | | |  |
| EP_1 | 4211801_19_C/T | 1A:474187796-474687796 | 12.90 | 0.23 | 59.1/52.1/53.7 |  | See QTL PIVNDFD_1 | See QTL PIVNDFD_1 | |
| EP_2 | 8530946_28_G/A | 5B:645663357-646503357 | 6.17 | 0.10 | 58.9/50.8/51.5 | WCSS1_contig10912959_5BL-4833  (grain yield) | TraesCS5B02G472200 | Oxidoreductase activity | |
|  |  |  |  |  |  |  | TraesCS5B02G472300 | NA | |
|  |  |  |  |  |  |  | TraesCS5B02G472400 | Protein binding  (protein ubiquitination) | |
|  |  |  |  |  |  |  | TraesCS5B02G472500 | Protein binding  (protein ubiquitination) | |
|  |  |  |  |  |  |  | TraesCS5B02G472600 | Protein binding  (protein ubiquitination) | |
|  |  |  |  |  |  |  | TraesCS5B02G472700 | Protein binding  (protein ubiquitination) | |
|  |  |  |  |  |  |  | TraesCS5B02G472800 | NA | |
|  |  |  |  |  |  |  | TraesCS5B02G472900 | 3-oxoacyl-[acyl-carrier-protein] reductase (NADPH) activity, oxidoreductase activity, NAD binding  (fatty acid biosynthetic process) | |
| EP_3 | 10514469_23_C/G | NA | 6.95 | 0.13 | 58.2/55.2/59.5 |  | NA | NA | |
| EP_4 | 36892695_26_C/G | NA | 8.00 | 0.16 | 60.8/56.1/58.2 |  | NA | NA | |
| *EP_5* | *4209538_61_C/T* | *5R:610604285-611704285* | *11.36* | *0.27* | *59.9/56.6/56.7* |  | *SECCE5Rv1G0339760* | *DNA-binding transcription factor activity*  *(regulation of transcription, DNA-templated)* | |
|  |  |  |  |  |  |  | *SECCE5Rv1G0339770* | *DNA-binding transcription factor activity, protein dimerization activity*  *(regulation of transcription, DNA-templated)* | |
|  |  |  |  |  |  |  | *SECCE5Rv1G0339780* | *DNA-binding transcription factor activity, protein dimerization activity*  *(regulation of transcription, DNA-templated)* | |
|  |  |  |  |  |  |  | *SECCE5Rv1G0339790* | *(DNA synthesis involved in DNA repair, DNA replication)* | |
|  |  |  |  |  |  |  | *SECCE5Rv1G0339800* | *Catalytic activity, phospholipase A2 activity, lipase activity, lysophosphatidic acid acyltransferase activity*  *(lipid homeostasis, fatty acid homeostasis,*  *phospholipid homeostasis, triglyceride homeostasis)* | |
|  |  |  |  |  |  |  | *SECCE5Rv1G0339810* | *Protein binding* | |
|  |  |  |  |  |  |  | *SECCE5Rv1G0339820* | *NA* | |
|  |  |  |  |  |  |  | *SECCE5Rv1G0339830* | *Serine-type carboxypeptidase activity*  *(proteolysis)* | |
|  |  |  |  |  |  |  | *SECCE5Rv1G0339840* | *NA* | |
|  |  |  |  |  |  |  | *SECCE5Rv1G0339850* | *Serine-type carboxypeptidase activity*  *(proteolysis)* | |
|  |  |  |  |  |  |  | *SECCE5Rv1G0339860* | *Protein binding* | |
|  |  |  |  |  |  |  | *SECCE5Rv1G0339870* | *NA* | |
|  |  |  |  |  |  |  | *SECCE5Rv1G0339880* | *Nucleic acid binding, RNA binding* | |
|  |  |  |  |  |  |  | *SECCE5Rv1G0339890* | *Serine-type carboxypeptidase activity*  *(proteolysis)* | |
|  |  |  |  |  |  |  | *SECCE5Rv1G0339920* | *Nucleic acid binding, RNA binding* | |
|  |  |  |  |  |  |  | *SECCE5Rv1G0339930* | *Nucleic acid binding, RNA binding* | |
|  |  |  |  |  |  |  | *SECCE5Rv1G0339940* | *Serine-type carboxypeptidase activity*  *(proteolysis)* | |
|  |  |  |  |  |  |  | *SECCE5Rv1G0339950* | *Protein binding* | |
|  |  |  |  |  |  |  | *SECCE5Rv1G0339960* | *Nucleic acid binding, RNA binding* | |
|  |  |  |  |  |  |  | *SECCE5Rv1G0339980* | *Protein binding* | |
|  |  |  |  |  |  |  | *SECCE5Rv1G0340000* | *Nucleic acid binding, RNA binding* | |
|  |  |  |  |  |  |  | *SECCE5Rv1G0340040* | *DNA-binding, DNA-binding transcription factor activity*  *(regulation of transcription, DNA-templated, ethylene-activated signaling pathway)* | |

**References**

1. Baron VS, Juskiw PE, Aljarrah M. Triticale as a forage. In: Eudes F, editor. Triticale. Springer, Cham; 2015. p. 189–212.

2. De Zutter A, De Boever J, Muylle H, Roldán-Ruiz I, Haesaert G. In vitro digestibility as screening tool for improved forage quality in triticale. F Crop Res. 2023;301:article 109009.

3. De Zutter A, Landschoot S, Vermeir P, Van Waes C, Muylle H, Roldán-Ruiz I, et al. Variation in potential feeding value of triticale forage among plant fraction, maturity stage, growing season and genotype. Heliyon. 2023;9:article e12760.
